# Play-based interventions for mental health: A systematic review and meta-analysis focused on children and adolescents with autism spectrum disorder and developmental language disorder

**DOI:** 10.1177/23969415211073118

**Published:** 2022-02-28

**Authors:** Gill Francis, Emre Deniz, Carole Torgerson, Umar Toseeb

**Affiliations:** Department of Education, 152532 University of York, York YO10 5DD, USA

**Keywords:** Play-based interventions, autism spectrum disorder, developmental language disorder, mental health, systematic literature review

## Abstract

**Background and aims:**

Play-based interventions are used ubiquitously with children with social, communication, and language needs but the impact of these interventions on the mental health of this group of children is unknown. Despite their pre-existing challenges, the mental health of children with developmental language disorder (DLD) and autism spectrum disorder (ASD) should be given equal consideration to the other more salient features of their condition. To this aim, a systematic literature review with meta-analysis was undertaken to assess the impact of play-based interventions on mental health outcomes from studies of children with DLD and ASD, as well as to identify the characteristics of research in this field.

**Methods:**

The study used full systematic review design reported to the Preferred Reporting Items for Systematic Reviews and Meta-analyses (PRISMA) guidelines (PRISMA prisma-statement.org) with pre-specified inclusion criteria and explicit, transparent and replicable methods at each stage of the review. The study selection process involved a rigorous systematic search of seven academic databases, double screening of abstracts, and full-text screening to identify studies using randomised controlled trial (RCT) and quasi-experimental (QE) designs to assess mental health outcomes from interventions supporting children with DLD and ASD. For reliability, data extraction of included studies, as well as risk of bias assessments were conducted by two study authors. Qualitative data were synthesised narratively and quantified data were used in the metaanalytic calculation.

**Main contribution:**

A total of 2,882 papers were identified from the literature search which were double screened at the abstract (n  =  1,785) and full-text (n  =  366) levels resulting in 10 papers meeting the criteria for inclusion in the review. There were 8 RCTs and 2 QEs using 7 named play-based interventions with ASD participants only. Meta-analysis of 5 studies addressing positive mental health outcomes (e.g. positive affect and emotional functioning) found a significant overall intervention effect (Cohen's d = 1.60 (95% CI [0.37, 2.82], p = 0.01); meta-analysis of 6 studies addressing negative mental health outcomes (e.g., negative affect, internalising and externalising problems) found a non-significant overall intervention effect (Cohen's d = 0.04 -0.17 (95% CI [-0.04, 0.51], p = 0.88).

**Conclusions:**

A key observation is the diversity of study characteristics relating to study sample size, duration of interventions, study settings, background of interventionists, and variability of specific mental health outcomes. Play-based interventions appear to have a beneficial effect on positive, but not negative, mental health in children with ASD. There are no high quality studies investigating the efficacy of such interventions in children with DLD.

**Implications:**

This review provides good evidence of the need for further research into how commonly used play-based interventions designed to support the social, communication, and language needs of young people may impact the mental health of children with ASD or DLD.

## Introduction

Autism spectrum disorder (ASD) and developmental language disorder (DLD) are common neurodevelopmental disorders, with prevalence rates of ∼1% and ∼7%, respectively (Baird et al., 2006, Norbury et al., 2016). The disorders are characterised by social, communication, and or language impairments. Young people with ASD and DLD also tend to have poorer mental health compared to their neurotypical peers (Yew & O’Kearney, 2013). Given their language and communication impairments, these young people may find it difficult to access talking therapies. There is some evidence from observational studies that play may be *associated* with a decrease in subsequent mental health difficulties in children with ASD and DLD (e.g. Toseeb et al., 2020a). However, observational studies cannot demonstrate whether such associations are causal. There is a growing body of experimental research reporting on mental health outcomes for young people with social, communication, and or language impairments from play-based interventions but the findings are inconsistent. Therefore, this systematic review and meta-analysis sought to review the evidence from experimental studies to investigate the *effectiveness* of play-based interventions for mental health in children and adolescents with ASD and DLD.

### ASD and DLD

ASD and DLD are common neurodevelopmental disorders. Both can be conceptualised as spectrum disorders such that affected individuals have a unique set of strengths and weaknesses. ASD is characterised by difficulties in social communication and interaction and restricted and repetitive behaviours, interests, and activities (American Psychiatric Association, 2013). These features of ASD manifest differently across affected individuals to varying frequencies and severities. Young people with DLD are impaired in their ability to learn and use oral language (Bishop et al., 2017). This is in the absence of certain biomedical conditions, such as ASD and hearing loss. The term DLD has been adopted in recent years and encompasses other terms such as specific language impairment (SLI). Young people with SLI have impaired oral language ability but their non-verbal cognitive ability is within the normal range (Tomblin et al., 1997). The term DLD does not require a discordant verbal and non-verbal cognitive ability; therefore, all young people with SLI can be described as having DLD. For consistency and ease of comprehension, the term DLD is used here to include both SLI and DLD. Additionally, young people with ASD and DLD tend to have poorer mental health compared to neurotypical young people.

### Mental health

Mental health is a multi-dimensional complex construct, which consists of both positive and negative features. Positive mental health refers to positive affect and has been described in the literature as happiness, wellbeing, and life-satisfaction (Westerhof & Keyes, 2010). Negative mental health refers to the presence of negative affect and behaviours and is the focus of most mental health research, commonly referred to as mental health disorders. These include symptoms of depression, anxiety, and conduct problems (American Psychiatric Association, 2013). The World Health Organisation defines health as “a state of complete physical, mental, and social wellbeing and not merely the absence of disease or infirmity” (World Health Organisation, 1948), suggesting that both positive and negative mental health are important features of overall mental health. The absence of mental health difficulties is not synonymous with positive mental health. For example, young people may be neither happy nor experiencing low mood, whilst others with a high level of mental health difficulties might have high levels of life-satisfaction (Patalay & Fitzsimons, 2018). This is in line with psychological theory, whereby positive and negative mental health are related but distinct constructs (Dual-continua model of mental health, Westerhof & Keyes, 2010). Given also that the correlation between positive and negative mental health is moderate (Patalay & Fitzsimons, 2016), the evidence suggests that positive and negative mental health should be considered separately.

Diagnosable mental health conditions are not necessarily the same as mental health difficulties. Experiencing mental health difficulties, such as symptoms of low mood, anxiety, irritability etc., are a normal part of life. Low mood is to be expected after a negative life event and anxiety is an adaptive response to alert us to imminent danger. These responses turn into a diagnosable disorder when they become persistent and cause functional impairment^1^ (American Psychiatric Association, 2013). Psychiatric diagnoses rely on individuals to be able to explain their symptoms and the related functional impairment. Young people with ASD and DLD are likely to struggle with this due to their language and communication difficulties. In addition to this, individuals who fall slightly below diagnostic thresholds may be precluded from a diagnosis. Such binary categorisations are unhelpful in understanding the frequency and intensity of mental health difficulties. For these reasons, symptom-based measures of mental health may be preferred as they provide a comprehensive account of the nature and type of difficulties rather than simply the presence or absence of disorder. This is important because some interventions may lead to an improvement at a symptom level, which improves the quality of life in the affected individuals.

Child and adolescent mental health can be viewed through the lens of social and emotional development. Features of mental health may manifest differently through development. During the first few years of life, an infant's ability to form relationships, recognise and respond to emotions, explore their environment, and meet major milestones may be considered a positive indicator of their mental health. Difficulties in these areas might sometimes be precursors to mental health difficulties in later life. As infants grow older and become children and then adolescents, it becomes easier to identify their feelings and behaviours as mental health difficulties. This is partly due to more established measures of mental health at these ages but also because behaviour at these ages more closely corresponds to documented symptoms of mental health difficulties. Therefore, in this systematic review, a broad definition of mental health has been adopted to account for the diversity in the manifestation of mental health difficulties across development.

### ASD, DLD, and mental health

Young people with ASD and DLD tend to have poorer mental health compared to their unaffected peers. Early research reported that approximately 70% of young people with a *language disorder* have a diagnosable mental health condition (Cantwell & Baker, 1987). This also translates at the symptom level. Young people with DLD have more symptoms of internalising and externalising problems compared to their unaffected peers (Yew & O’Kearney, 2013). There is a similar state of affairs for young people with ASD. Approximately 40% of children and adolescents with ASD have a diagnosable anxiety disorder (van Steensel et al., 2011). They also have higher rates of a diagnosable depression disorder (Hudson et al., 2019). In addition to the quantitative differences in mental health difficulties in ASD and DLD populations, there is also emerging evidence to suggest that they experience such difficulties in a qualitatively different way. For example, young people with ASD experience sensory symptoms differently (Ben-Sasson et al., 2009) and have different coping strategies (Mazefsky et al., 2013).

The co-occurrence of an ASD or DLD and mental health difficulties may be due to three possible explanations: 1) The presence of ASD or DLD features lead to mental health difficulties, 2) mental health difficulties lead to ASD or DLD, and or 3) ASD or DLD are caused by a third factor (e.g., genetics or a common environmental risk factor). First, the presence of ASD or DLD affects children's cognitive abilities which, according to the social information processing theory (Crick & Dodge, 1994), affect their social interactions. Young people with ASD or DLD may be less able to navigate social situations leading to withdrawal and the onset of psychopathology. For example, young people with DLD have higher levels of social anxiety (Durkin et al., 2017), which is associated with poorer mental health compared to their neurotypical peers In addition to this, it is well documented that young people with ASD or DLD are more likely to be bullied by siblings and peers (Toseeb et al., 2018, 2020b, van den Bedem et al., 2018), and may have poorer quality friendships (e.g., Andres-Roqueta et al., 2016), all of which are associated with mental health difficulties. The second possibility is that earlier social and emotional difficulties exacerbate or trigger the onset of features of ASD or DLD. Usage-based approaches to social, language, and communication development highlight the importance of social context (e.g., Tomasello, 2003). Social interactions between primary caregiver and child, and also between the child and their peers, provide a context in which to develop social, language, and communication skills (e.g., Hoff, 2006). Young people with pre-existing social and emotional difficulties may find it difficult to access these social interactions and so features of ASD or DLD are exacerbated. The final possibility is that the occurrence of ASD or DLD and mental health difficulties is due to a third unmeasured factor. Recent evidence suggests that common environmental factors, primarily consisting of socioeconomic variables, explain much of the variation in social, communication, and mental health outcomes (Bignardi et al., 2021). Additionally, common genetic variants may influence both DLD and mental health (Newbury et al., 2019, Toseeb et al., 2021). Playing with others may be beneficial for the mental health of children and adolescents with ASD or DLD as it enables them to learn and practise key emotional and behavioural regulation skills.

### Play

Play is the leading form of activity in most children's lives which provides them with limitless opportunities to satisfy their unrealisable tendencies and sudden desires (Vygotsky, 1933). Play is a difficult term to define, as any activity that is freely chosen, child-driven, and pleasurable for the child can be conceptualised as play (Sturgess, 2009). The overall characteristics of play are described as spontaneous, free, non-literal, intrinsically motivated, pleasurable, and purified from externally imposed rules (Rubin, 1982; Saracho & Spodek, 1998; Hughes, 2003).

Numerous theories have tried to explain the necessity of play in children's lives. *Home Ludens*, a modern historical theory of play, suggests that play is the core element of children's lives as it provides them freedom, pleasure, and joy (Huizinga, 1949). In addition, the constructivist theory indicates that play is an integral part of children's development, as it is closely related to cognitive and language skills (Piaget, 1952). From a psychosocial perspective, it is claimed that play is children's own way to self-define social reality and provides them with a suitable environment to build their self-control skills (Erikson, 1993). In psychoanalysis, it is proposed that play creates a fantasy world for children to cope with the complexity of reality and provides the opportunity to explore and express their deep emotional states (Freud, 1955). As suggested by these theories, far from being thought to be a trivial activity in children's lives, play has a fundamental role in supporting children's various development, including, but not limited to, their cognitive, emotional, language and behavioural regulation skills.

The term *play-based intervention* stands for either the socio-cognitive techniques that are specifically built on the elements of play or the implementations that are delivered during the playtime or within the play settings (Ingersoll & Walton, 2013). The importance of play-based interventions in supporting children's development was recognised in the early 20^th^ century. *Little Hans* is known as the first child who was treated with play for his phobic symptoms (Freud, 1909). Melanie Klein (1929), a student of Freud, also used play as a therapeutic tool to apply Freud's psychoanalytic techniques initially used with adults to subsequently apply to children. Wells (1912) and Lowenfeld (1939) were the other earliest child psychoanalysts that used sand play to support children with emotional problems. However, Virginia Axline (1947), a student and later colleague of Carl Rogers, firstly used the term *play therapy* and introduced client-centred play therapy as a structured play intervention to treat children's emotional difficulties.

Children of today's world are growing up in a dynamic environment in which they may not be getting enough time and opportunities to play freely. Today, children are playing less than those in previous generations; Natural England (NE) reports that less than 10% of the new generation play outside, naturally, whereas this rate was more than 50% in their parents’ generation (England Marketing, 2009). In the United States, it is claimed that children's playtime is significantly reduced by their parents stealing their spare playtime to invest more in schoolwork (Chudacoff, 2007). The lack of play in children's lives, thus, may be the reason underlying the failure of the new generation of children in reaching some developmental milestones at certain ages. For instance, early opportunities for make-believe play are suggested to be crucial for developing imaginative thought (Goldstein, 1994). It is also suggested that a child's playfulness is connected to their secure attachment, emotion regulation, empathy, and emotional resilience (Whitebread, 2017). Play deprivation in early childhood, however, is suggested to be associated with emotional/self-dysregulation and aggression with higher deprivation results in higher prevalence of dysfunctioning in these areas (Brown, 2014). In addition, children with identifiable deficits in play skills are at further risk in accessing developmental affordances from play (Toseeb et al., 2020b).

Numerous interventions have been built on play to support children's social, emotional, and cognitive development. However, the characteristics of such interventions vary in the sense of the definition and representation of play, play approach (e.g., directive or non-directive), or type of play (make-believe, fantasy, parallel, etc) that the delivered intervention is built on (Thomas & Smith, 2004). It is crucial to well-define play to differentiate the interventions that are solely built on play from any other interventions. *Play* is defined as activities that are *intrinsically motivated, spontaneous, free from externally imposed rules, guided by organism-guided questions, non-literal and requires active engagement* (Rubin et al., 1983, p.698). The authors also drew a clear line between play and game as: “Play is further distinguished from game in that the latter phenomenon is goal oriented whereas the former phenomenon is not. One plays for the satisfaction of playing. One engages in games to compete, to win, and to achieve some specified goal” (p.728). Taking Rubin et al.'s broad definition of play into account, this review focuses on the interventions that were solely play-based, delivered during the playtime or within the play settings such as, robot/animal-based play (e.g., canine-assisted play), peer-mediated play (e.g., the SENSE Theatre), parent-child interaction play (e.g. DIR/Floortime), school-based play (e.g., Early Start Denver Model), non-directive play (e.g., child-centred play), cognitive-therapeutic play (e.g., Jungian play), etc.

Although there is considerable evidence supporting the effectiveness of play-based interventions on children's holistic development, the majority of interventions have focused on children's social, communication, and language skills. For example, a study on the relationship between play and children's social development has reported that social-interactive play increases social competence in young children (Newton & Jenvey, 2011). Another study reports a significant increase in social interaction and language skills and a decrease in play deficits and disruptive behaviours of children with special educational needs and disabilities following a play intervention (O’Connor & Stagnitti, 2011). Furthermore, it has been suggested that play increases receptive and expressive vocabulary in at-risk preschoolers (Han et al., 2010), and a strong link has been found between symbolic play skills and functional language abilities of children with ASD (Mundy et al., 1987). Despite this, very few empirical studies have targeted the mental health outcomes of children. A recent systematic review of child-led play interventions also argued that such interventions mostly target children's social development instead of focusing on emotional outcomes (Gibson, et al., 2017).

The effectiveness of play-based interventions on children's emotional outcomes has only grabbed a few researchers’ interests. For example, one study investigated the effectiveness of unstructured play in reducing the anxiety level of young children in paediatric inpatient care and reported that a nurse-delivered play intervention was highly effective in reducing the anxiety levels of hospitalised children (Al-Yateem & Rossiter, 2017). Additionally, another study examined whether an unstructured play intervention reduced the stress level of hospitalised children in inpatient care and reported that children aged 7 to 11 years old showed a significant decrease in cortisol levels (a marker for stress) compared to the control group (Potasz et al., 2013). In addition, a playful approach of Six Bricks and DUPLO® has also been found effective on positive emotions and emotional competence of early adolescents (Harn & Bo, 2019).

### Relevant systematic literature reviews

There are a handful of published reviews and meta-analysis synthesising work that address the mental health of individuals with social, communication and or language impairments generally or assess the impact of a play-based intervention on such individuals’ mental health. For instance, in a meta-analysis of cross-sectional studies reporting the prevalence of depressive disorders among individuals with ASD, Hudson, Hall, and Harkness (2019) reported that lifetime and prevalence of depressive disorders was 14.4% (95% CI 10.3–19.8) and 12.3% (95% CI 9.7–15.5), respectively, across samples of children, adolescents, and adults with ASD. Their findings indicate that the rates of depressive disorders are high among individuals with ASD. In a separate narrative review, Blewitt et al. (2019) investigated the extent to which preservice education programmes led by early childhood teachers and carers address ASD children's social and emotional development. After screening papers from MEDLINE Complete, PsychINFO and ERIC between January 1999 and July 2019, the authors identified only a limited number of studies (n  =  19) and concluded that teachers are not adequately prepared to deal with ASD children's social, emotional and behavioural challenges. Newton and Jenvey (2011) recommended that programmes should promote naturalistic and embedded social skill instruction within and across everyday interactions, play, activities and the environment, thereby offering contextually relevant opportunities to strengthen children's social-emotional skills can help.

Three additional review studies were found that synthesise the effect of different types of play-based intervention - Child-Centered Play-therapy (Hillman, 2018), DIR/Floortime (Mercer, 2017), and Pivotal Response Treatment (PRT) (Verschuur, et al., 2014). These are recognised play-based interventions derived from play-therapy approaches developed to support ASD children's social, emotional, and cognitive development and the reviews included in their synthesis at least one mental health outcome. Child-centered play therapy is used to improve core concerns related to ASD, such as social communication skills, joint attention and emotional regulation (Hillman, 2018). The effectiveness of Child-centered play therapy with ASD populations was investigated by Hillman (2018) in a comprehensive search of the Educational Resources Information Center (ERIC) and PsycINFO databases between 1900 and September 2017. Only four studies were identified in this review, of which only two addressed mental health outcomes - emotional growth and empathy, using single-case and multiple-baseline designs, with mixed results. According to Hillman (2018) more research is needed to understand the impact of Child-centered play therapy on ASD children. The other review, Mercer (2017), assessed the plausibility of, and evidentiary support for, the treatment DIR/Floortime - an intervention that uses therapeutic goals to work with autistic children and typically involves a functional emotional assessment along with other social communication and language evaluations. Mercer (2017) searched the Academic Search Complete, PsycINFO, and PubMed databases articles up to January 2015 and identified ten studies of which two studies used experimental designs and included an assessment of ASD children's emotions as a study outcome. Mercer concluded that although there is some support for the effectiveness of the DIR/Floortime intervention; the support is weak because of design flaws. The third review used PRT to teach pivotal behaviours to children with ASD in order to achieve generalised improvements in their functioning on four aspects of pivotal functioning: (a) motivation, (b) self-initiations, (c) responding to multiple cues, and (d) self-management (Verschuur et al., 2014). The authors screened studies published in ERIC, Linguistics and Language Behavior Abstracts, Medline, PubMed, and PsychINFO databases between December and June 2013 and identified thirty five intervention studies of which three studies reported mental health outcomes (increase in positive affect).

Whereas, the first two meta-analyses highlighted mental health challenges as prevalent among individuals with ASD none of the reviews of play-based interventions explicitly focused on the impact of mental health outcomes. This indicates a gap in the literature and a need for a review of play-based interventions and mental health outcomes.

### The current study

A better understanding of how interventions also impact the mental health of children is valuable for evaluating the utility of these interventions in fostering holistic child development in neurodiverse populations. This review is important as it illuminates how play-based interventions designed for children with social, communication, and or language impairments related to diagnoses of ASD and DLD impact mental health outcomes. Questions addressing both the contextual design and the effectiveness of interventions can validate good practice in the field or make recommendations for improvement. This systematic review and meta-analysis addresses the following research questions:

1. What are the characteristics of play-based interventions used to support children and young people with ASD or DLD to improve their mental health outcomes?
2. Are play-based interventions designed for children with ASD or DLD effective in supporting their mental health?

## Methods

A systematic literature review with meta-analysis was conducted following standards from the Cochrane Handbook for systematic reviews and the Preferred Reporting Items for Systematic reviews and Meta-Analyses (PRISMA) guidelines for evaluating and reporting on the effectiveness of interventions (Higgins et al., 2021; Page et al., 2021). This involved searching for, locating, quality appraising and synthesising, both narratively and statistically, all the relevant studies that can address the question about the effectiveness of play-based interventions on the social, communication, language, and mental health outcomes of children with social, communication, and language impairments. The systematic review included studies exploring all outcomes in a broad ‘mapping’ of the field but narrowed to an in-depth synthesis and a meta-analysis of studies with the findings for mental health outcomes only. At the inception of the review, *a-priori* criteria for including studies in the review were developed in a protocol through collaborative discussions among the study authors before commencing the search process to identify potentially relevant studies.

### Eligibility criteria

Pre-specified inclusion criteria were developed to identify studies evaluating high-quality interventions based on topic, study designs, characteristics of participants and intervention and control conditions, in order to include studies that can address the research questions. These pre-specified inclusion criteria provided the basis for selecting studies for inclusion in the review (see Table 1).

**Table 1.** Criteria for Selecting Studies for this Review.

Study Criteria	Inclusion	Exclusion
Design	Experimental Designs	Experimental designs using single group comparisons
	RCT	
	non-equivalent group quasi-experimental designs (pre/post test design)	
	regression discontinuity design,	
	time-series designs	
Date	Studies published between 2000–2021	Studies published < 2000
Language	Studies published in English	Non-English publications
Participants	Participants age ranges include:	Adults
	Early years (0-7yrs)	
	Middle childhood (8-12 yrs)	
	Adolescence (13-19 yrs)	
	Classification of participants with social-communication needs e.g.:	
	DLD	
	Autism	
	Asperger's Syndrome	
	SLI	
	Social-communication disorder	
Interventions	Play interventions addressing mental health as well as social, communication, and language outcomes in children with social-communication impairments	Non-play-based interventions
Comparisons	Comparison of play-based interventions to a control group using no intervention, wait-list control, usual care, or an active control group which is a nonplay-based intervention.	Comparisons of two play-based interventions
Outcomes	Mental-Health/Wellbeing outcomes	Non social-communication, socio-emotional or mental health outcomes
	Happiness	
	Self-esteem	
	Wellbeing	
	Depression	
	Anxiety	
	Stress	
	Internalising/Externalising/Total difficulties	
	Emotional difficulties	
	Social - Communication outcomes	
	Speech, Language, and communication skills	
	Language Learning outcomes	
	Friendships (peer engagement)	
	Prosociality (empathy)	
	Social skills (play skills)	
Setting	Research settings e.g.	No exclusions on the basis of description of research settings.
	Schools	
	Clinical settings	
	Research settings	
	Play intervention setting e.g.:	
	Therapy centre	
	School	
	Home	
	Other	
	Play intervention provider e.g.	
	Play therapists	
	Psychologists	
	Teachers	
	Parents	
	Peers	
	Researchers	
	Animal/Robot/Canine assisted	

Only studies using experimental designs were included. These include: (1) randomized controlled trials (RCTs): studies that randomly assign participants to intervention and control or comparison conditions; (2) quasi-experimental designs (QEs) using pre/post- tests designs with non-equivalent comparison groups where participants are not randomly assigned to conditions; (3) regression discontinuity designs which assign participants to conditions on the basis of a predetermined cut-off on a continuous variable; and (4) time-series designs involving at least three observations made both before and after a treatment. All included studies were required to have a control or comparison group that either did not receive a treatment or were on a waitlist to receive treatment after the end of the experiment (Shadish, Cook, & Campbell, 2002). As the aim was to evaluate intervention designs that infer causality, studies that employed single case experimental designs and multiple-baseline designs were excluded. Although they are commonly used in research with children with social, communication, and language disorders and can provide limited evidence of causality, for the purposes of this review the inclusion criteria specified only the most robust designs were included.

In addition, as mentioned earlier, this review focuses on the studies that used interventions that were inherently playful but not based on any other behavioural modification techniques. Therefore, studies that focus on behavioural interventions, teaching or training, peer-based group activities, and game-with-rules (e.g., script-fading, activity schedules, group activity schedules, computer/online-games, video-prompting, behavioural intervention, wearable devices/virtual reality, and gaming activities) were kept out of the scope of this review. Hence, to meet this inclusion criterion, interventions should either be solely play-based or delivered during the playtime or within the play settings. Also, studies that compared the effectiveness of two or more play-based interventions on the mental health outcome of children with ASD or DLD were also excluded.

An initial scoping exercise was conducted by the first and second authors to inform the aspects of the study's eligibility criteria. The scoping exercise was especially useful for two reasons: (1) to ascertain the period during which a significant record of publications reporting play-based interventions with children with social-communication impairments exist so as to specify the time-line for the review, and; (2) to identify relevant terms, specifically the name of play-based interventions and terms used to refer to children with ASD and DLD. The list of play-based interventions identified include: Play Therapy, Theraplay, Play-based interventions, PRT, Joint Attention, Symbolic Play, Engagement & Regulation (JASPER, Advancing Social-communication And Play (ASAP), LEGO Therapy, The Developmental, Individual Difference, Relationship-Based (DIR) Floortime Model, The Paediatric Autism, Communication Therapy (PACT), Stay, Play, & Talk, PLAY Project, Project ImPACT, Early Start Denver Model (ESDM), E-PLAYS, CCPT (Child-centred play therapy), Filial therapy, Animal/Parent/Peer/Sibling assisted play, Non-named play-based interventions. Classification terms used to refer to individuals with social-communication needs were also identified.

Only relevant publications dating from the last two decades (2000 to 2021) published in English Language journals were included. The scoping exercise indicated the majority of play-based experimental interventions were undertaken during that timeline. We screened for peer-reviewed journal articles as well as masters and doctoral theses but excluded conference papers and grey literature due to insufficient resources.

To identify eligible studies, detailed specifications of the population, intervention, comparators, outcomes, and settings (PICOS) were outlined as key components of interest (Thomas et al., 2021). Study participants were inclusive of children diagnosed with a social, communication, and language impairment including ASD or DLD. Participants’ ages ranged from infancy to adolescence and we categorised infancy as less than two years, childhood from 2 to 12 years, and adolescents 12 to 21 years (Hardin et al., 2017). The intervention types considered are all play-based interventions whether using validated or less formally developed programmes, as well as those supporting children with social-communication disorders through peer-mediated and parent-mediated designs. Comparisons between intervention groups are based on studies having the following control conditions: no intervention; wait-list control; usual care; or interventions with active controls if the comparison is a nonplay-based control. Studies with outcomes relating to the social communication, language, and mental health needs of children were deemed eligible for inclusion. Studies needed to describe the setting of the intervention in detail as this level of description is important for understanding the context under which play-based interventions may or may not be effective.

### Information sources

To identify studies, a comprehensive search of academic research databases was undertaken between January and March 2021. The searchable publication databases used were: ‘Ebscohost/ERIC’, ‘Web of Science’, ‘Pubmed’, ‘SCOPUS’, ‘PsycINFO’, ‘PROQuest’, and ‘ETHOS’.

### Search strategy

A consistent search strategy was applied across databases. Boolean search strings were developed using keywords. These included references to play as a generic term stringed with types of language impairments e.g., Play AND “language disorder”, Play AND “specific language impairment”, etc or types of play-based interventions, only e.g. “PRT”, or “Theraplay”. The full list of Boolean search strings is presented in Appendix A.

### Selection process

An exhaustive search of the selected academic databases using all combinations of the Boolean search strings was undertaken by one author [ED] and all hits were downloaded into the Mendeley Reference Manager software (Mendeley, 2021). Systematic reviews and meta-analyses based on the same topic area generated from the search were retained to undertake manual hand-searching of their reference lists as a strategy for capturing additional peer-review articles not published in the databases searched. The titles and abstracts of all studies in Mendeley were independently double screened and the papers which both authors [ED & GF] agreed met the eligibility criteria were carried over for independent double screening of full texts and agreement. To identify any other potential articles overlooked from the database searches, backward chaining was done by authors [ED & GF] by consulting the reference lists of papers recommended for full-text screening when in-text citations indicate an article might be highly relevant.

### Data extraction process

A data extraction form was designed to abstract relevant bibliographic details and information about the sample and sample characteristics, intervention and control or comparison conditions, and quantified outcomes from individual papers included in the review. To quality assure the robustness of the completion of the data extraction form for each included study, the following process was followed: first, all four authors [ED, GF, CT, UT], extracted data from one paper and the results were compared for consistency and sufficiency of the evidence to answer the research questions; subsequently, data extraction was divided between three authors [ED, GF, CT] such that data from each paper was independently extracted by two different authors and compared for agreement, with any adjustments being made if there was any initial disagreement, and arbitration by the fourth author [UT] where necessary.

### Outcomes

The review investigated the effect of play-based interventions on mental health and wellbeing outcomes including variables like happiness, self-esteem, wellbeing, depression, anxiety, stress, emotional difficulties, internalising or externalising difficulties, etc. All post-intervention outcomes were extracted. The decision was taken to include both primary and secondary outcomes, and experimenter-developed outcome measures as well as validated measures, where applicable.

Mid-intervention data and post-intervention follow-up data were excluded. Other pertinent data extracted include information about participant characteristics, e.g., social communication and language diagnosis, age of participants, etc., and intervention characteristics e.g., sample size, the background of the person delivering the intervention, setting of the intervention, etc. Missing data were sourced by contacting the corresponding author of a given study, if necessary.

### Study risk of bias

An assessment of the risk of bias^2^ was conducted for all studies included in the review using sample questions from the revised ‘Cochrane Risk of Bias Tool for Randomized Trials’ (RoB 2) and ‘Cochrane Risk of Bias Tool in Non-randomized Studies of Interventions’ (ROBINS-1) to appraise RCTs and QE studies, respectively (see Appendix B). Both instruments focus on different aspects of trial design, conduct and reporting comprising six domains under which signal questions are posed to elicit information about features of the trial that are relevant to the risk of bias (Sterne et al., 2016, 2019). The six domains addressed the risk of bias arising from:

- randomisation in RCT or confounds in QEs
- deviations from the intended interventions in RCTs or effect of assignment to interventions in QEs
- missing outcome data
- measurement of the outcome
- selection of the reported result

In each domain, a judgement of ‘low’, ‘high’, or ‘some concerns’ for risk of bias is asserted and used to make an overall judgment about the ‘risk of material bias’ of the study. A judgement of ‘high’ risk of bias for any individual domain will lead to the result being at ‘high’ risk of bias overall, and a judgement of ‘some concerns’ for any individual domain will lead to the result being at ‘some concerns’, or ‘high’ risk, overall. The aim is to expressly identify issues that are likely to affect the ability to draw reliable conclusions from the study (Sterne et al., 2019). The shortened versions of the risk of bias tools used were trialled by all four authors on a selected paper and a final version was agreed upon through discussion. The review papers were appraised independently by the first and second authors [GF & ED] and 25% of each author's sample was independently appraised by the other two authors [CT & UT].

### Effect measures

Effect sizes with 95% confidence intervals were computed based on post-intervention measurements as study outcomes were continuous. Effect sizes were calculated based on Cohen's *d* using the online Practical Meta-analysis Calculator and replicated in Stata (StataCorp, 2021. Standardised mean difference and standard error values for each outcome were generated from values of means and standard deviations reported in the individual papers. . Where data were not available, the effect size was computed using other appropriate statistical indices, e.g. F-test statistics and sample sizes. Issues of missing data were addressed by contacting the corresponding author of individual papers to request additional information. According to White, Redford, & MacDonald (2020), suggested thresholds of statistically significant effects of Cohen's *d* in social science research include: (1) 0 < d < 0.1 indicates a trivial effect; (2) 0.1 < d < 0.2 indicates a small effect; (3) 0.2 < d < 0.5 indicates a moderate effect; (4) 0.5 < d < 0.8 indicates a medium size effect; (5) 0.8 < d < 1.3 indicates a large effect; (6) 1.3 < d < 2.0 indicates a very large effect; (7) d > 2.0 indicates an extremely large effect.

It was necessary to correct for differences in the direction of measurement scales because the standardised mean difference method does not account for such differences (Higgins, et al., 2021). This was necessary for studies grouped as measuring negative mental health. For example, some scales increased with trait severity (e.g., a higher score indicated severe negative mental health) whilst others decreased (e.g., a lower score indicated less severe negative mental health). To ensure that all scales pointed in the same direction, the mean values for scales with positive directionality were multiplied by −1, before standardisation (Higgins, et al., 2021).

To account for statistical dependencies, cases where the data are dependent i.e., multiple outcomes from one study, the average effects were computed to yield a single effect estimate (Borenstein, 2009). Some studies were comparisons of two intervention approaches; hence, these were included only if it was a comparison between a play-based and non-play-based intervention. Therein, the non-play-based intervention was treated as the control group and the play-based approach as the intervention group.

### Synthesis methods

Study outcomes were classified based on whether the measures assessed positive or negative mental health. In addition, both narrative and statistical analyses were undertaken. The narrative analysis involved comparing intervention characteristics across studies [RQ1] and statistical analysis involved meta-analytic calculations of the mental health outcomes to make inferences about the effectiveness of play-based interventions [RQ2]. To undertake statistical syntheses of the review data, meta-analyses of study outcomes were conducted in Stata using the inverse variance estimation method and a random effects model. The random-effects model assumes the existence of within-and between-study variability or heterogeneity in estimating individual and combined study effects and attempts to minimise both sources of variance (Borenstein, 2009). The meta-analytic results are depicted with forest plots which show effect sizes and corresponding confidence intervals for studies at the individual and combined level, as well as heterogeneity statistics. Within study variability is assessed by the χ2 (Q) test and a significant p-value (or a large χ2 statistic relative to its degree of freedom) is indicative of evidence of heterogeneity of intervention effects (Hedges, 1982; Higgins et al., 2021). Between-study variance is inferred from tau (T²). The proportion of variance in study effect size is indicated by I² (Higgins & Thompson, 2002). Values of 25%, 50%, and 75% are proposed as benchmarks of low, moderate, and high variance with large I² inviting speculation for the reasons of the variance (Higgins, et al., 2003; Borenstein, 2009). A high I² suggests that variability between effect size estimates are due to between-study differences rather than the sampling variation (Borenstein et al., 2017). Also, the prediction interval or range of predicted effects (standard deviation of the overall effect size estimate) is reported. However, this should be interpreted cautiously, as the results can be very spuriously wide or spuriously narrow when the number of included studies are small (Higgins et al., 2021). Further inspection of the data included assessment of publication bias to explore concerns around the under-reporting of non-significant results. This involves visual inspections of funnel plots to check for study asymmetry and statistical tests of small study effects using the non-parametric trim and fill method to assess the impact of missing studies from potential publication bias. A sensitivity analysis to compare studies by research design or risk of bias results was undertaken. Meta-regression analyses were undertaken to investigate whether study heterogeneity could be explained by study moderators or pre-specified study characteristics depending on whether there are sufficient numbers of homogenous studies.

## Results

### Study selection results

The automated searching of electronic databases yielded 2882 papers including existing related systematic review papers used for citation searches and additional sources obtained from backward chaining, that is, articles identified from checking the reference list of review papers and from following up on relevant citations flagged during the full-text screening (n  =  59). After deduplication, 1785 papers were retained. Titles and abstracts were double screened by the first and second authors [GF & ED]. The two authors [GF & ED], agreed on the inclusion of 366 papers, the exclusion of 1362 papers, and identified 42 papers that could not be evaluated due to being inaccessible^3^ at the time of screening. There was disagreement on 15 papers that were discussed and resolved through discussion with the research team. Cohen's kappa coefficient for inter-rater reliability was κ  =  .88 (95% CI, .85 - .91) which indicates that the agreement between the first two authors on the selection of articles for full-text screening was very good.

**Figure 1.**
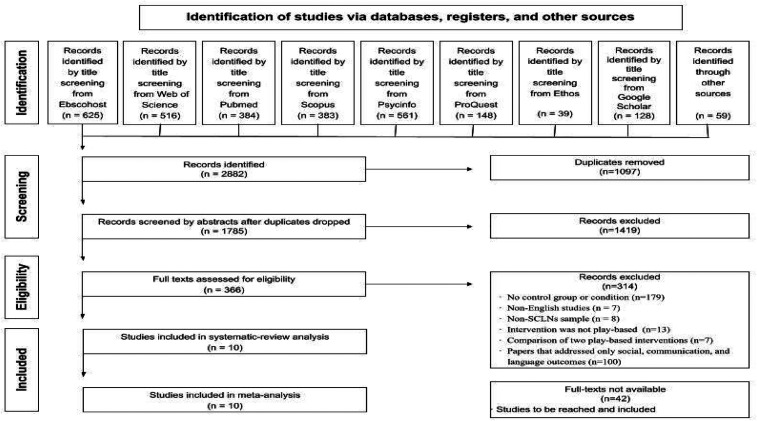
PRISMA Flow Diagram for Record of Systematic Searches of Databases

The process of double screening was repeated on the 366 papers eligible for full-text screening. Through a process of consensus agreement, a total of 314 papers were excluded for the following reasons: a) no control or comparison group (n  =  179); b) published in languages other than English (n  =  7); c) sample participants did not have a diagnosed social communication or language impairment (8); (d) intervention was not play-based (n  =  13); e) comparison between two play-based interventions (n  =  7); f) only social, communication, and language outcomes (n  =  100). In total, 10 papers met the eligibility criteria, i.e., explicitly reported mental health outcomes for study participants which is the focus of this review (see PRISMA Flow Diagram in Figure 1).

Of particular note was the fact that the 10 studies were published during the period 2008 to 2021, although the database screening was conducted from the period January 1st, 2000 to January 31st, 2021. The absence of records of play-based interventions using RCTs and QEs addressing mental health outcomes of children with ASD or DLD before 2008 is striking.

### Study characteristics

The characteristics of the ten studies are described based on study design, sample sizes, and characteristics of play-based interventions used. A full description of studies at the individual level is presented in Table 2.

**Table 2.** Included Studies and Study Characteristics

Study ID	Study reference, publication, and country	Sample characteristics	Research design	Play intervention	Outcome variable	Summary of results
S01	Corbett et al. (2017) Publication: *Journal of Autism* Country: USA	Sample size: N = 30 Sample: Children with ASD Age: 8-14 years old Girls: N = 6 (20%) Boys: N = 24 (80%) INV: N = 17 CON: N = 13	Design: RCT Type of control: Randomly allocated, waitlist control Control group treatment: SENSE Theatre® Outcome Measure: The STAI-C (Spielberger et al., 1983) Measure standardised: Yes	Name of intervention: SENSE Theatre® Intervention valid: Yes Type of play: Role play Place: Outside of school Provider: Not reported Duration: 40 hours	Reported outcome:Trait anxiety Category: Negative mental health	Test of between-subject effects revealed a significant group effect on post-STAI-C Trait, with pre-STAI-C Trait included as a covariate (F(1, 27) = 9.16, p = 0.005). Changes in play did not show a significant mediational effect on changes in trait-anxiety (B = −0.32; CI = −3.35 to 2.11). Conversely, the direct effect of the intervention on changes in trait-anxiety remained significant (B = −6.97, CI = −12.62 to −1.31).
					Reported outcome: State anxiety Category: Negative mental health	No group effect was observed for STAI-C State (F(1, 27) = 0.03, p = 0.86)).
S02	Doernberg et al. (2021) Publication: *Journal of Autism and Developmental Disorders* Country: USA	Sample size: N = 25 Sample: Children with HF ASD Age: 6-9 years old Girls: N = 3 (12%) Boys: N = 22 (88%) INV: N = 18 CON: N = 7	Design: RCT Type of control: Randomly allocated, waitlist control Control group treatment: Treatment as usual Outcome Measure: The APS (Fehr & Russ (2014)) Measure standardised: Yes	Name of intervention: Pretend Play Intervention valid: Yes Type of play: Pretend play Place: School Provider: The researchers Duration: 100 minutes	Reported outcome: Total positive affect Category: Positive mental health	Results did not indicate any significant effects for children’s abilities to generate a list of positive feelings, nor define complex emotions appropriately.
					Reported outcome: Total negative affect Category: Negative mental health	Results did not indicate any significant effects for children’s abilities to generate a list of negative feelings, nor define complex emotions appropriately.
S03	Ioannou et al. (2020). Publication: *Frontiers in Psychology* Country: USA	Sample size: N = 77 Sample: Children with HF ASD Age: 8-16 years old Girls: N = 18 (24%) Boys: N = 59 (76%) INV: N = 44 CON: N = 33	Design: RCT Type of control: Randomly allocated, waitlist control Control group treatment: SENSE Theatre® Outcome Measure: The STAI-C (Spielberger et al., 1983) Measure standardised: Yes	Name of intervention: SENSE Theatre® Intervention valid: Yes Type of play: Role play Place: Outside of school Provider: Not reported Duration: 40 hours	Reported outcome: State anxiety Category: Negative mental health	There was no difference in State anxiety between EXP and WCL groups [F(2,71) = 0.07, p = 0.935].
					Reported outcome: Trait anxiety Category: Negative mental health	Children in the EXP group reported significantly less Trait anxiety than children in the WLC group following intervention [F(2,71) = 6.87, p = 0.01].
S04	Pajareya & Nopmaneejumruslers (2011) Publication: Journal of *Autism* Country: Thailand	Sample size: N = 32 Sample: Children with ASC Age: 2-6 years old Girls: N = 5 (15%) Boys: N = 28 (85%) INV: N = 16 CON: N = 16	Design: RCT Type of control: Randomly allocated control group Control group treatment: Treatment as usual Outcome Measure: (1) The FEAS (Greenspan, DeGangi & Wieder, (2001). (2) The FEDQ (Pajareya, Sutchritpongsa & Sanprasath, 2014). Measures standardised: Yes	Name of intervention: DIR/Floortime Intervention valid: Yes Type of play: Parent and child interaction play Place: Home Provider: The first author Duration: 240 hours	Reported outcome: Functional Emotional Assessment Score (FEAS) Category: Positive mental health	The change of the FEAS score for the control group reflects the overall developmental progression of only 1.9 (SD = 6.1), compared to the increment of 7.0 (SD = 6.3) for the intervention group. The Student t test shows that the difference is statistically significant (p = .031).
					Reported outcome: Functional Emotional Developmental Score (FEDQ) Category: Positive mental health	Developmental rating of the children was estimated by the parent using the Thai version of the Functional Emotional Questionnaires at baseline and follow-up. The change in data for the intervention group shows that there was a more statistically significant gain in it than in the data of the control group.
S05	Rezaei et al. (2018) Publication: *Journal of Children* Country: Iran	Sample size: N = 34 Sample: Children with ASD Age: Mean = 12.36 Girls: N = 12 (35%) Boys: N = 22 (65%) INV: N = 17 CON: N = 17	Design: RCT Type of control: Randomly allocated, waitlist control Control group treatment: PRT + Risperidone Measures: The ABC (Akhondzadeh et al., 2010) Measures standardised: Yes	Name of intervention: Pivotal response treatment (PRT) (Koegel, 2011) + Risperidone Intervention valid: Yes Type of play: Parent and child interaction play Place: School Provider: Speech/language therapist Duration: 27 hours	Reported outcome: Irritability Category: Negative mental health	There was no significant difference between the INV and Control groups in Irritability subscale.
					Reported outcome: Hyperactivity Category: Negative mental health	There was no significant difference between the INV and Control groups in Hyperactivity subscale.
S06	Schottelkorb et al.. (2020) Publication: *Journal of Counseling & Development* Country: USA	Sample size: N = 23 Sample: Children with ASD Age: 4-10 years old Girls: N = 4 (17%) Boys: N = 19 (83%) INV: N = 12 CON: N = 11	Design: RCT Type of control: Randomly allocated, waitlist control Control group treatment: Treatment as usual Measure: CBCL (Achenbach & Rescorla, 2001). Measures standardised: Yes	Name of intervention: Child-centred play (Axline, 1947) Intervention valid: Yes Type of play: Non-directive play Place: Not reported Provider: Graduate-level counselling students and two licensed counsellors Duration: 12 hours	Reported outcome: Externalising problems Category: Negative mental health	Following the same trend as previous analyses, participants in the play therapy treatment group were reported to have decreased externalising symptoms from pre- to post-testing (M = 68.67, SD = 9.35; M = 63.08, SD = 7.90), whereas control group scores increased (M = 65.36, SD = 9.54; M = 67.27, SD = 8.72).
S07	Siller et al. (2014) Publication: *Journal of Autism and Developmental Disorders* Country: USA	Sample size: N = 70 Sample: Children with ASD Age: 2-6 years old Girls: N = 6 (9%) Boys: N = 64 (91%) INV: N = 36 CON: N = 34	Design: RCT Type of control: Randomly allocated control group Control group treatment: Parent Advocacy Coaching (PAC) Measures: (1) PCSB (Ainsworth, 1978) (2)AB (Ainsworth, 1978) (3) MPCA (Hoppes & Harris, 1990) Measures standardised: Yes	Name of intervention: Focused Playtime Intervention (Siller et al.,2013) Intervention valid: Yes Type of play: Parent and child interaction play Place: Research lab + participants’ home Provider: Trained graduate and postdoctoral students in developmental psychology and counselling. Duration: 18 hours	Reported outcome: Maternal Perceptions of Child Attachment (MPCA) Category: Positive mental health)	A significant main effect of treatment group allocation on gains in parent reported attachment behaviours (MPCA scores), t(48) = 3.0, p < .01.
					Reported outcome: Proximity/ Contact Seeking Behavior Scale (PCSB) Category: Positive mental health)	Proximity and Contact Seeking Behaviors was only marginally significant, t(54) = 1.8, p\.08.
					Reported outcome: Avoidant Behavior Scale (AB) Category: Positive mental health)	For children’s Avoidant Behaviors, results revealed a significant main effect of treatment group allocation on improvements in Avoidant Behaviors from Time 1 to Time 2, t(54) = 2.2, p\.05.
S08	Solomon et al. (2014) Publication: *Journal of Developmental and Behavioral Pediatrics* Country: USA	Sample size: N = 128 Sample: Children with ASD Age: 2-5 years old Girls: N = 20 (16%) Boys: N = 108 (84%) INV: N = 64 CON: N = 64	Design: RCT Type of control: Randomly allocated control group Control group received treatment: Treatment as usual Measure: The FEAS (Greenspan et al., (2001). Measures standardised: Yes	Name of intervention: PLAY Project home consultation programme (Solomon et al., 2007) Intervention valid: Yes Type of play: Parent and child interaction play Place: Home Provider: 6 PLAY consultants (1 occupational therapist, 2 speech and language therapists, and 3 special educators) Duration: 36 hours	Reported outcome: Functional Emotional Assessment Score (FEAS) Category: Positive mental health	The FEAS video ratings showed a significant moderate time 3 group effect with the PLAY group showing improvement in observed socioemotional behaviour, whereas the CS group remained stable.
S09	Duifhuis et al. (2017) Publication:*Journal of Autism and Developmental Disorders* Country: Netherlands	Sample size: N = 24 Sample: Children with ASD Age: 3-8 years old Girls: N = 4 (16%) Boys: N = 20 (84%) INV: N = 11 CON: N = 13	Design: QE Type of control: Non-randomly allocated control group Control group received treatment: Yes, treatment as usual Measure: Child Behavior Checklist (CBCL; Achenbach & Rescorla, 2001). Measures standardised: Yes	Name of intervention: Pivotal response treatment (PRT) (Koegel, 2011) Intervention valid: Yes Type of play: Parent and child interaction play Place: School Provider: Therapist Duration: 15 hours	Reported outcome: Internalizing score Category: Negative mental health	The analysis did not show any treatment effects on internalising score of the INV group on CBCL.
					Reported outcome: Externalizing score Category: Negative mental health	The analysis did not show any treatment effects on externalising score of the INV group on CBCL.
S10	Pilarz (2009) Publication: *PhD Dissertation* Country: USA	Sample size: N = 26 Sample: Children with ASD Age: 3-12 years old Girls: N = 5 (19%) Boys: N = 21 (81%) INV: N = 13 CON: N = 13	Design: QE Type of control: Non-randomly allocated control group Control group received treatment: No treatment Measure: The Functional Emotional Assessment Scale (FEAS) (Greenspan, DeGangi & Wieder, (2001). Measures standardised: Yes	Name of intervention: DIR/Floortime Intervention valid: Yes Type of play: Parent and child interaction play Place: Not reported Provider: Certified school psychologist Duration: 16 hours	Reported outcome: Functional Emotional Assessment Score (FEAS) Category: Positive mental health	The slopes of the pretest scores for the total scale score did not significantly vary across conditions; p-values ranged from .092 to .549.

#### Study designs

The 10 studies comprised RCTs (n  =  8) and QEs (n  =  2). Overall, RCT studies *randomly allocated participants* to intervention (n  =  224) and control (n  =  195) groups and QE studies *assigned participants* to the intervention (n  =  24) and control (n  =  26) groups non-randomly. In the majority of studies, participants received either no treatment and then the same treatment as the intervention group on a waiting list (n  =  5), treatment as usual (TAU, n  =  3), a different intervention (Parent advocacy coaching (PAC), n  =  1) or no treatment (n  =  1).

#### Sample sizes of sStudies

The sample size of the included studies ranged between 23 and 128 (mean sample  =  47), with a total of 469 participants. Intervention groups’ sample sizes varied from 11 to 64 with a total of 248 participants, while control groups’ samples were between 7 and 64 with a total of 221 children. Of the total sample, 386 were boys (82%) and 83 were girls (18%). Of the included studies, seven were focused on childhood with an age range of 2 to 12, while the other three studies focused on late childhood and adolescence with their samples’ ages ranging from 8 to 16.

#### Participant identification

All studies were conducted with children with ASD with two being focused on children with “high functioning” ASD. There was no study reporting any other type of social, communication, and or language impairment or used participants with multiple diagnoses. To confirm children's ASD diagnosis, eight out of the 10 studies used additional autism screening tools. The Autism Diagnostic Observation Schedule - Generic (ADOS-G, Lord et al., 2000) was the most commonly used measure as six of the included studies (Siller et al., 2014; Corbett et al., 2017; Solomon et al., 2014; Duifhuis et al., 2017; Ioannou et al., 2020; Doernberg et al., 2021) used this scale to test the autistic traits of their sample. ADOS-G is a gold standard, semi-structured, standardised observational tool administered by a clinician or researcher that focuses on children's social, communication, and cognitive skills. It consists of two domains, namely social and communication domains with four distinct sections which serve to diagnose children at different language levels. The ADOS-G is suitable to use with children from 2 years old to adulthood and the diagnostic classification relies on two different cutoff values, autism (cutoff  =  12) and ASD (cutoff  =  8) (Lord et al., 1999). In addition, Autism Diagnostic Interview-Revised (ADI-R, Lord et al., 1994) scale was also used by two studies to test the ASD characteristics of their samples (Siller et al., 2014; Doernberg et al., 2021). ADI-R is a semi-structured interview for parents/caregivers of children at risk for possible autism diagnosis. The scale is suitable to use with children from 18 months old to adulthood and consists of 93 items which focuses on three developmental domains: Language/ communication, reciprocal social interactions, and restricted, repetitive and stereotyped behaviours. The ADI-R cutoff values for autism diagnosis are as follows: Social interaction (cutoff  =  10), communication and language (cutoff  =  7–8) and restricted/repetitive behaviours (cutoff  =  3). Moreover, the Childhood Autism Rating Scale (CARS, Schopler et al., 1986), Social Responsiveness Scale–2nd Edition (SRS-2; Constantino & Gruber, 2012), and Early Social Communication Scale (ESCS, Seibert et al., 1982) were the other measures that were used in some studies, respectively; Pajareya and Nopmaneejumruslers (2011), Schottelkorb et al. (2020), and Siller et al. (2014).

#### Characteristics of play-based interventions

A range of different types of play-based interventions were used in the included studies, such as parent-child interaction play (n  =  6), role/pretend play (n  =  3), and non-directive play (n  =  1). Although nine studies applied *named play-based interventions* (SENSE, DIR/Floortime, PRT, PLAY Project, CCPT, FPI), only one study used a *non-named play intervention* (pretend play, see S02). Interventions’ frequencies ranged from once a month to 4 times a week, and the total duration of the interventions ranged from 100 min to 240 h. Three interventions were conducted at participants’ schools, two interventions were conducted at home, two interventions were undertaken outside of school (exact locations were not reported) and one intervention was conducted at a research lab; nevertheless, two papers did not indicate the intervention place. Intervention providers were licensed counsellors (n  =  3), therapists (n  =  2), researcher/s (n  =  2), and not reported (n  =  3). Detailed information about the characteristics of the play-based interventions is provided in Table 3.

**Table 3.** Characteristics of the Play-Based Interventions

Name of the intervention	Type of Play and Leadership Roles	Target age, developmental areas and skills	Core Features of the intervention	Study ID
The Developmental, Individual-Difference, Relationship-Based (DIR / Floortime®)	Type of Play: Parent-child interaction play Provider: Therapist Role: Trains the parent Leader: Child Role: Leads the play Mediator: Parent Role: Joins the child's play and supports them according to the therapist's feedback.	Specifically developed for children with ASD: Yes Age: Early childhood Developmental Area: Functional and Emotional Development Targeted skills: 1-Auditory processing 2-Visual–spatial processing 3-Tactile processing 4-Motor planning 5-Sensory modulation	The DIR / Floortime is a developmental, individual-difference, and relationship-based approach that was introduced by (Greenspan & Wieder (1997). It is a child-centered, parent-mediated play intervention that aims to support children's social functioning, and emotional and language development. The DIR / Floortime intervention targets children in their early childhood years who show delay in their developmental progression and provides them a range of support according to their developmental needs (Pajareya and Nopmanejumruslers, 2011). In the DIR / Floortime intervention, first, the parent(s) attend initial training sessions about the principles of DIR/Floortime. During sessions, the therapist trains the parents and other individuals, who play a role in the child's everyday life, about how to set a playground in their home to play with their children, how to extend parent-child interaction their play, and increase circles of social communication and interaction between them (Pilarz, 2009)	S04, S10
The Pivotal Response Treatment (PRT)	Type of Play: Parent-child interaction play Provider: Therapist Role: Trains the parent Leader: Child Role: Leads the play Mediator: Parent Role: Joins the child's play and supports them according to the therapist's feedback.	Specifically developed for children with ASD: Yes Age: Early childhood Developmental Area: Social and Emotional Development Targeted skills: 1- Motivation 2- Self-initiation, 3- Self-Management 4- Responsiveness to multiple cues 5- Empathy	The PRT is a play-based intervention that was developed by Koegel et al., (1987) to increase motivation while teaching new skills to children with ASD. It targets five pivotal skills of children with ASD: motivation, self-initiation, self-management, and responsiveness to multiple cues, and empathy (Koegel et al., 1987). The PRT has been shown effective in improving social, communication skills and reducing behaviour problems in children with ASD (Rezai et al., 20018). In the PRT sessions, first, the therapist teaches parents how to use the PRT techniques while the child gets used to the therapist. Second, the therapist and parents discuss the developmental needs of the child and set the therapy goals. Third, the parents start to play with their children using the PRT techniques under the therapist's supervision.Fourth, the therapist explains the other possible ways that the parents can apply the PRT techniques into the daily life activities at home. Lastly, the therapist gives the parents videotaped and written feedback about how to improve their PRT skills and apply them in a different range of activities (Duifhuis, 2017).	S05, S09
The Play and Language for Autistic Youngsters (PLAY) Project Home Consultation	Type of Play: Parent-child interaction play Provider: Therapist Role: Trains the parent Leader: Child Role: Leads the play Mediator: Parent Role: Joins the child's play and supports them according to the therapist's feedback	Specifically developed for children with ASD: Yes Age: Early childhood Developmental Area: Social and Functional Development Targeted skills: 1- Social reciprocity 2- Social competency 3- Child's autism symptomatology (autistic traits)	The PLAY Project was developed by Solomon et al. (2007) based on the DIR / Floortime approach (Greenspan & Wieder, 1997) which aims to help the parents to connect with their own child with ASD. The PLAY is a child-centred, parent-mediated intervention that targets to improve social development and reduce autism symptomatology of children with ASD. In the PLAY, the therapist/consultant trains the parent about how to interact with their child with ASD “*through coaching, modelling, and video feedback. During coaching, consultants help parents identify their child's subtle and hard to detect cues, respond contingently to the child's intentions, and effectively engage the child in reciprocal exchanges”* (Solomon et al., 2014, p. 478).	S07
The Focused Playtime Intervention (FPI)	Type of Play: Parent-child interaction play Provider: Therapist Role: Observes, guides, models, and provides feedback to the parent Leader: Child Role: Plays independently Mediator: Parent Role: Joins the child's play and guides and supports the child's social responsiveness and communication attempts	Specifically developed for children with ASD: Yes Age: Early childhood Developmental Area: Social - Communication Development, Attachment Targeted skills: 1- Attachment skills 2- Social responsiveness 3- Child's spoken communication skills	The FPI is a home-based naturalistic intervention that was developed by Siller et al. (2013). It aims to provide a naturalistic playground to the child to understand the child's developmental stage to set parents’ goals and plan a play environment that would support communication and attachment behaviours of the child (Siller et al., 2014). The FPI is a family-centred, parent-mediated intervention that targets responsive parental communication to improve participatory and attachment skills of the child in a naturalistic play setting in their homes. In the FPI, parent advocacy coaching sessions hold in their homes, in which the consultant helps the parents to understand their child's developmental needs, guides them to initiate functional and meaningful social interactions during the play with their child, and trains them about how to respond their child's social-communication attempts in a naturalistic way during their playtime (Siller et al., 2013).	S08
The Social Emotional NeuroScience Endocrinology Theatre® (SENSE)	Type of Play: Role / Pretend play Provider: Therapist Role: Trains the peers to support the child with ASD during their role play Leader: Child Role: Demonstrates role play Mediator: Peers Role: Join the child's play to encourage and motivate their friend with ASD, and provide a warm social environment to them.	Specifically developed for children with ASD: Yes Age: Early childhood Developmental Area: Social, Cognitive and Emotional Development Targeted skills: 1- Imagination 2- Theory of mind 3- Social competence	The SENSE is a theatre-based play intervention that was developed by Corbett et al. (2011) and targets social and emotional functioning in children with ASD. The SENSE is designed as a peer-mediated intervention that aims to improve social competence and reduce the stress level of children with ASD with an intensive peer support. SENSE includes theatrical games, role-playing, and exercises in which children work on their roles for the play (Corbett et al., 2017). In the SENSE, peers receive training on ten core principles (Corbett et al., 2014) that are mainly about how to socially support the child with ASD, provide them with a warm social environment, and engage in positive social interactions with them. Following the training, peers and the child with ASD engage in role-playing activities under the guidance of the interventionist. While performing role-play, children with ASD experience positive social interactions, develop their level of imagination and TOM skills, learn how to engage with their peers, and respond to social invitations (Ioannou et al., 2020).	S01, S03
The Child-Centered Play Therapy (CCPT)	Type of Play: Non-directive play Provider: Therapist Role: Joins the child during their play, follows the child's leadership, and reflects the child's emotions. Leader: Child Role: Leads the play Mediator: None	Specifically developed for children with ASD: No Age: Early childhood Developmental Area: Social, Emotional and Behavioural Development Targeted skills: 1- Attachment skills 2- Emotional regulation 3- Emotional recognition 4- Self-control 5- Self-regulation 6- Social competence	The CCPT is a naturalistic and therapeutic intervention that was firstly introduced by Axline (1947) based on Carl Rogers's client-based psycho-therapy principles. It is a theoretically grounded intervention that targets children's emotional and behavioural problems. The CCPT is a child-centered, non-directive approach that aims to help children to explore and express their deep feelings, emotions, and thoughts during a free play session. During the CCPT, the therapist develops a warm and friendly relationship with the child, accept the child unconditionally, provide them a suitable atmosphere to freely express their feelings, reflect their feelings, believe in the child's problem-solving potential, does not direct or limit the child unless it is necessary (Axline, 1969, p.73). In the CCPT, the therapist creates a play environment that would suit CCPT principles. At the beginning of the session, the therapist informs the child about the time of the play. During the play, the therapist follows the child's lead and takes notes about the play themes, play behaviours, verbalisations, expressed emotions and informs the parents accordingly after each session (Schottelkorb et al., 2020).	S06
Pretend Play	Type of Play: Pretend / Symbolic play Provider: Therapist Role: Joins the child during their pretend-play activity, follows the child's leadership, and reflects the child's emotions. Leader: Child Role: Leads the play Mediator: None	Specifically developed for children with ASD: No Age: Early childhood Developmental Area: Social, Emotional and Cognitive Development Targeted skills: 1- Imagination 2- Perspective taking 3- Affect expression 5- Social communication 6- Prosociality	Pretend play is a common type of play that is closely related to a range of cognitive abilities of a child such as symbolic functioning, memory, and imagination. Children start pretend-playing (make-believe) at around 2, and become masters in pretend play at around 4 years of age (Piaget, 1952). The Pretend play intervention is a child centred play intervention that targets the child's imaginative skills, expressed emotions, verbalisation, and role-play skills. In the pretend play intervention, in which the therapist creates a free play environment with unstructured toys for the child. During the intervention, the therapist joins the child's play, follows the child's lead, uses prompts, and reflects the child's emotions to help children to developing new skills such as making up new things, creating stories, and to improve their level of imagination and organisation (Doernberg et al., 2021).	S02

#### Mental health outcomes

Of the included studies, five studies used parent-report, three used self-report, and four used observational scales to test the effectiveness of the play-based interventions on the mental health outcome of children with ASD. In terms of measuring the negative mental health outcomes, the researchers used the State-Trait Anxiety Inventory for Children (STAI-C, Spielberger et al., 1983) to measure the anxiety level of children with ASD. In addition, the Child Behaviour Checklist (CBCL, Achenbach & Rescorla, 2001) was used to measure internalising and externalising problems, the Aberrant Behavior Checklist (ABC, Aman et al., 1985) was used to test irritability and hyperactivity, and the Kusche Affective Inventory-Revised (KAI-R, Kusche et al., 1988) was used to measure total negative affect. In terms of testing the positive mental health outcomes; however, the Functional Emotional Assessment Scale (FEAS, Greenspan et al., 2001) was used to assess emotional functioning; the Functional Emotional Developmental Questionnaire (FEDQ, Greenspan & Greenspan, 2002) was used to assess emotional development; the Proximity and Contact Seeking Behaviors (PCSB, Ainsworth, 1978) and Maternal Perceptions of Child Attachment Scales (MPCA, Hoppes and Harris, 1990) were used to evaluate children's attachment-related behaviours; and the KAI-R scale was used to measure total positive affect in children with ASD. The measures noted are well-used standardised measures of the relevant concepts. More information about the mental health classifications of the reported outcomes and the outcome measures can be found in Appendix D.

#### Geographical location of studies

Studies were undertaken in the United States of America (n  =  7), Thailand (n  =  1), Netherlands (n  =  1), and Iran (n  =  1).

### Risk of bias

Included RCT and QE studies were appraised separately and independently by four raters (all four study authors) using a risk of bias tool appropriate to each study design. The results of the risk of bias ratings are reported for RCTs (n  =  8) and QEs (n  =  2) separately and presented in Table 4 and Table 5, respectively. Of the eight RCT studies, one study (S02) did not meet the random allocation criterion whereas the other seven randomly allocated the participants into the intervention and control groups. Although the allocation process was concealed in the majority of the RCTs (S01, SO2, SO3, S06, S07, S08), two studies did not conceal the allocation sequence (S04 & S05). Only one of the studies (S08) clearly reported that participants were blind to the intervention; three studies reported that carers and interventionists were blind to the intervention (S01, S03, & S05). In one study (S02), the number of missing observations was high which means the reported outcome data were not available for all or nearly all participants. Additionally, five studies reported having a pre-specified analysis plan (S01, S04, S05, S06, & S08), and three studies had a priori protocols (S03, S05, & S06). No studies reported selected outcome variables and all RCTs used appropriate measures to report the outcome variables. In summary, of the eight RCTs, one study was judged to have a high risk of bias (S02), five demonstrated some concerns (S01, S03, S04, S07, S08) and two was judged to have a low risk of bias (S05 & S06).

**Table 4.** Risk of bias assessment of the RCT research

	Domain 1			Domain 2			Domain 3			Domain 4		Domain 5				Overall Risk of Bias
Study ID	1.1 Random allocation?	1.2 Allocation sequence concealed?	1.3 ROB	2.1. Participants blinded to the intervention?	2.2. Carers/interventionists blinded to the intervention?	2.3 ROB	3.Outcome data available for all participants?	3.2 Bias due to Missing outcome?	3.3 ROB	4.1 Inappropriate method to measure the outcome?	4.3 ROB	5.1 Pre-specified analysis plan?	5.2 Selected reported outcome?	5.3 A priori protocol?	5.4 ROB	Summary ROB
S01	Yes	Yes	Low	NI	PY	SC	Yes	No	Low	No	Low	Yes	No	No	Low	SC
S02	No	PY	SC	NI	NI	SC	No	NI	SC	No	Low	No	PN	N	High	High
S03	Yes	PY	Low	PN	PY	SC	Yes	PN	Low	No	Low	No	No	PY	SC	SC
S04	Yes	PN	Low	NI	NI	Low	Yes	No	Low	No	Low	PY	N	N	SC	SC
S05	PY	PN	Low	PN	PY	Low	Yes	PN	Low	No	Low	PY	PN	PY	Low	Low
S06	Yes	PY	Low	No	PN	Low	Yes	PN	Low	No	Low	PY	PN	Yes	Low	Low
S07	Yes	PY	Low	No	No	Low	PY	Y	SC	No	Low	PN	No	No	SC	SC
S08	Yes	Yes	Low	PY	PN	SC	PY	PN	Low	PY	Low	PY	PN	PN	SC	SC

**Table 5.** Risk of bias assessment of the QE research

	Domain 1			Domain 2			Domain 3			Domain 4		Domain 5				Overall Risk of Bias
Study ID	1.1 Confounding effect of intervention?	1.2 Participants selected based on their characteristics?	1.3 ROB	2.1. Intervention group clearly defined?	2.2. 2.4 Intervention groups defined before the intervention?	2.3 ROB	3.1 Outcome data available for all participants?	3.2 Bias due to Missing outcome?	3.3 ROB	Inappropriate method to measure the outcome?	4.3 ROB	5.1 Pre-specified analysis plan?	5.2 Selected reported outcome?	5.3 A priori protocol?	5.4 ROB	Summary ROB
S09	PN	PN	Low	Yes	PY	Low	Yes	PN	Low	No	Low	PY	PN	Yes	Low	Low
S10	PY	No	High	Yes	Yes	Low	PY	PN	Low	No	Low	PY	No	No	SC	High

Of the two QE studies (S09 & S10) none allocated the participants into the intervention group based on their observed characteristics after the intervention started. However, the authors identified a potential confounding effect of the intervention in one study (S10), which posed a high risk of bias. Both QE studies clearly defined their intervention groups based on the information recorded before the intervention. Additionally, outcome data were available for all participants in both studies and none reported selected outcomes or potential bias due to missing outcomes. Both studies used appropriate methods to measure their outcomes and had a pre-specified analysis plan; however, only one of the QE studies had a priori protocol (S09). Consequently, of the QE studies, one was judged to have a low risk of bias (S09) and the other demonstrated a high risk of bias (S10).

### Results of synthesis

Of the ten studies which met the criteria for inclusion in the review, all had sufficient numerical data to be included in the meta-analysis. Eighteen mental health outcomes were identified and categorised as either positive mental health (n  =  10) or negative mental health (n  =  8). Average scores were computed in cases where an individual study had multiple outcomes in the same category (either positive or negative mental health), resulting in fewer outcomes; positive mental (n  =  5) and negative mental health (n  =  6) outcomes.

#### Intervention effects

**Figure 2.**
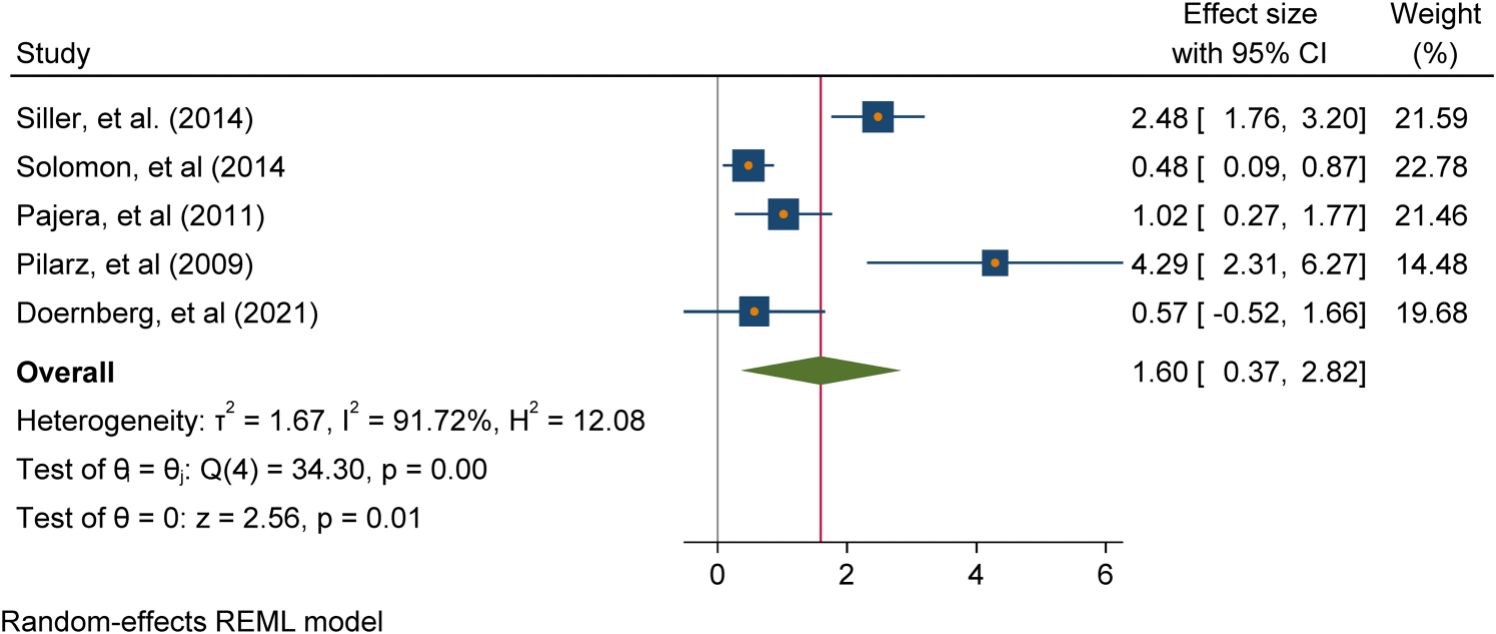
Forest Plot Showing Effect of Positive Mental Health Outcomes is 1.60 (95% CI [0.37, 2.82], p = .01).

**Figure 4.**
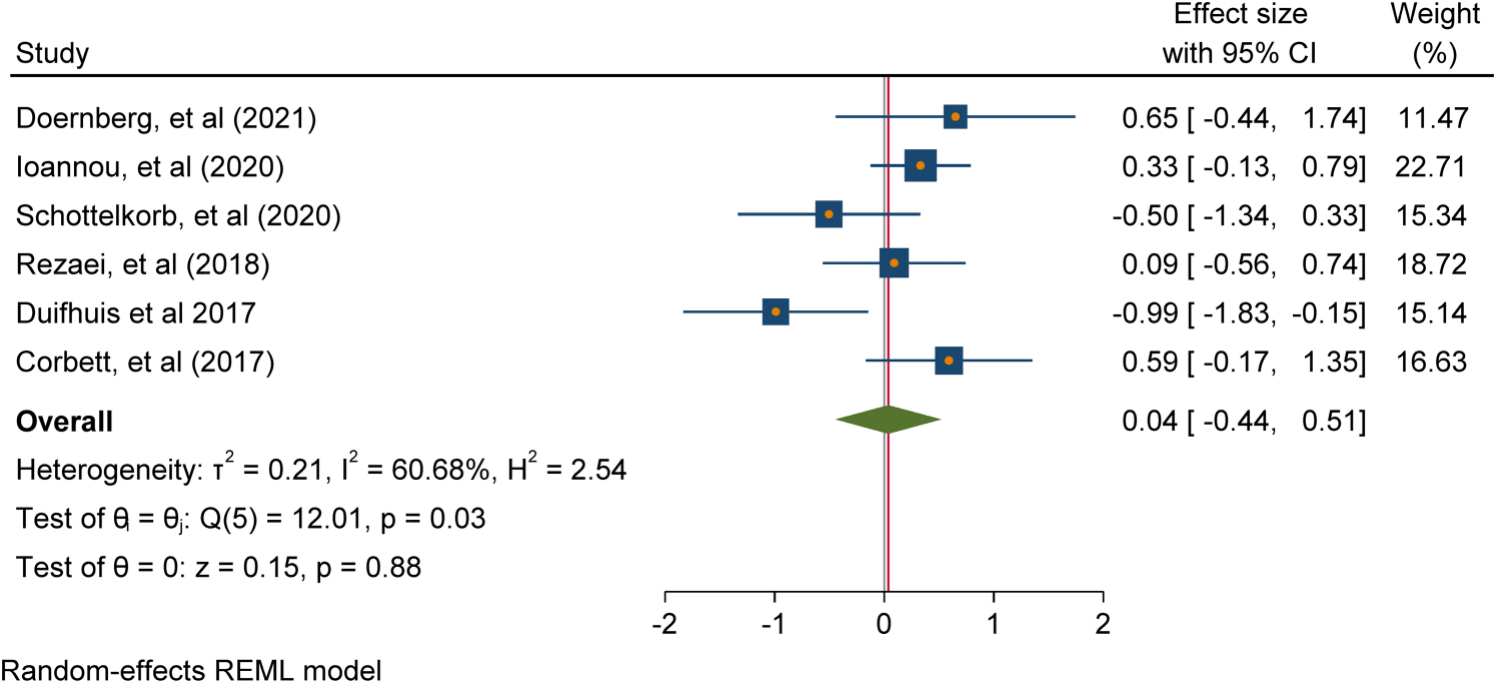
Forest Plot Showing Effect of Negative Mental Health Outcomes is 0.04 (95% CI [-0.44, 0.51], p = .88).

The effect of play-based interventions on positive and negative mental health outcomes are discussed and the results of the meta-analysis are displayed in the forest plots. The forest plots (see Figure 2 and Figure 4) show the study labels and corresponding effect size estimate represented by a blue square with an orange circle or marker at the centre of the square that indicates the study-specific effect size with respective horizontal lines that show the magnitude of the confidence intervals. Generally, longer lines indicate wider confidence intervals which suggest smaller samples. The absence of a horizontal line from some boxes indicates the study has restricted confidence intervals which may suggest more precise effect sizes (Higgins, et al., 2021). The black vertical line running through zero, known as the line of no effect, indicates differences between significant and non-significant interventions. The red vertical line shows the overall measure of the effect. All intervention effects in this sample were statistically significant.

**Figure 3.**
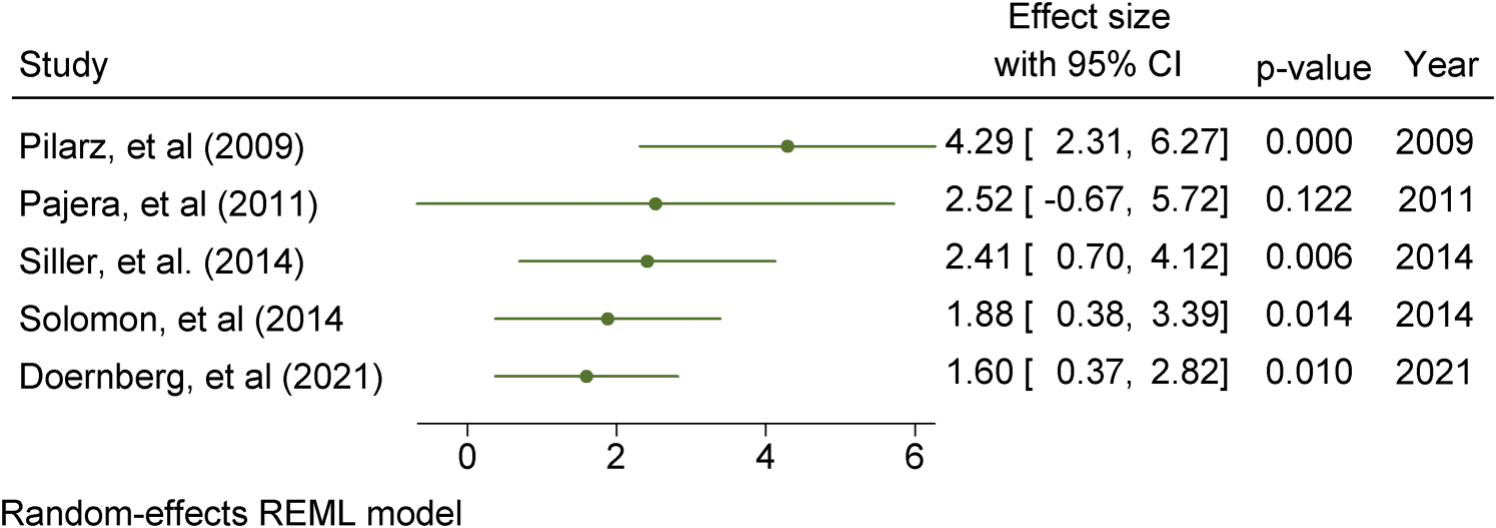
Cumulative Forest Plot Showing Effect of Positive Mental Health Outcomes by Year.

Play-based interventions intervening on positive mental health outcomes for children with ASD had an overall significant positive effect of 1.60 (95% CI [.37, 2.82], p  =  .01). The results are shown in the forest plot in Figure 2. There is evidence of significant statistical heterogeneity, Q(4)  =  34.30, p < .001 and high proportional variance, I²  =  91.72% which confirms observations of variability in study characteristics. The predicted interval of intervention effects ranges between −5 and 5 but this should be treated cautiously given the small number of studies. Interestingly, there is a distinct pattern of a decrease in the size of study effects from 2008 to the present (Figure 3).

**Figure 5.**
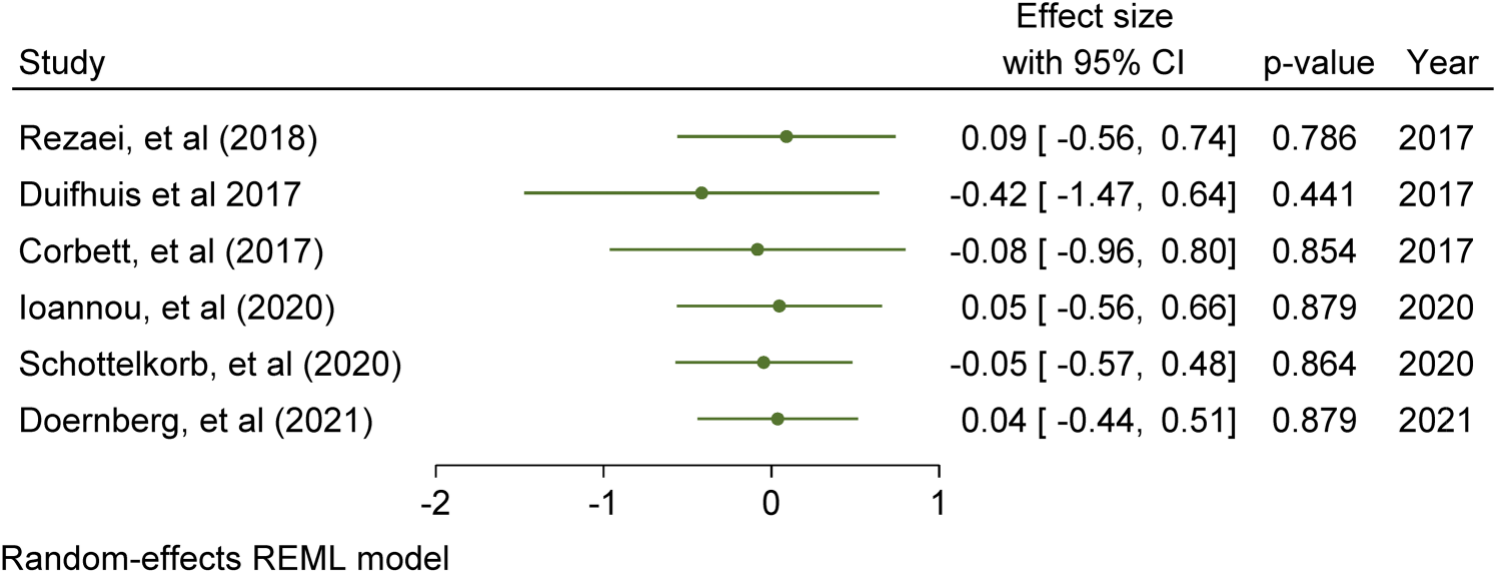
Cumulative Forest Plot Showing Effect of Negative Mental Health Outcomes by Year

In contrast, interventions addressing negative mental health outcomes show overall intervention effects were not significant of .04 (95% CI [-.44, 0.51], p  =  .88). In addition, there is significant study heterogeneity Q(5)  =  12.03, p  =  .003 and high proportional variance, I²  =  60.68%, which confirms observations of variability in study characteristics with the predicted interval range between −2 and 2. The results are shown in the forest plot in Figure 4. The pattern of change in intervention effects in progressive years of research is sporadic for studies researching negative mental health outcomes (see Figure 5).

#### Small-Study effects

**Figure 6.**
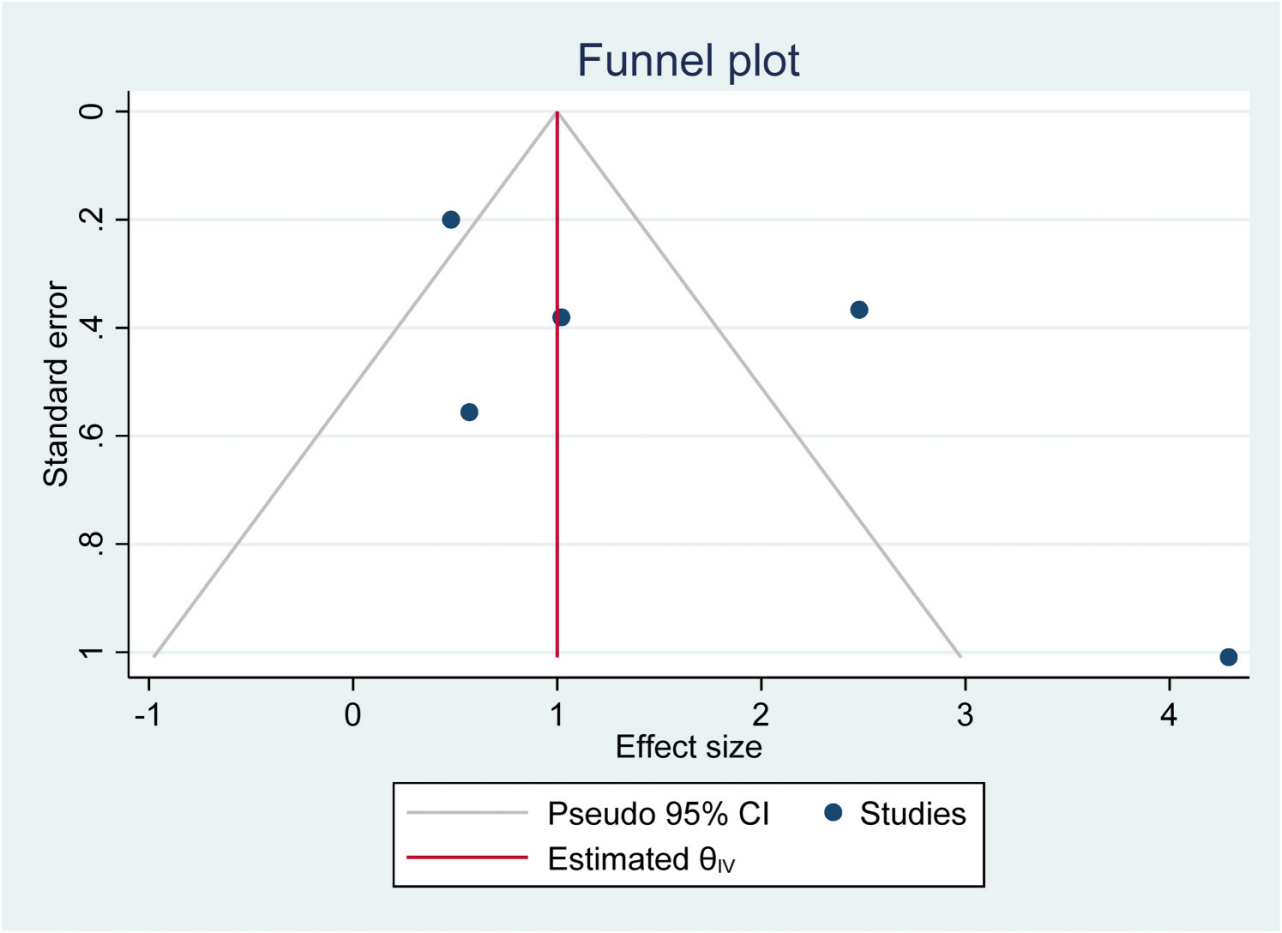
Funnel Plot of Positive Mental Health Outcomes.

**Figure 7.**
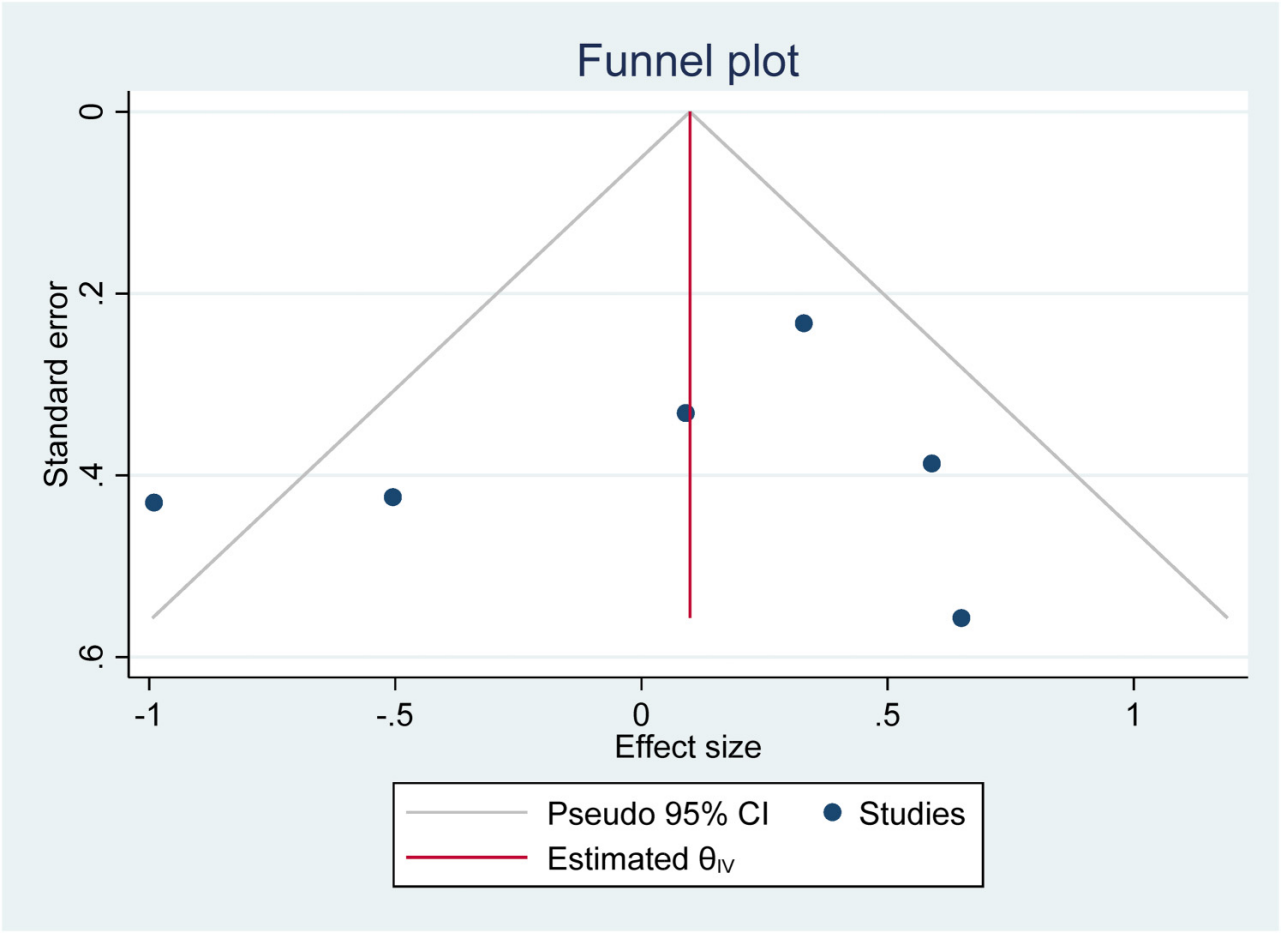
Funnel Plot of Negative Mental Health Outcomes.

From scrutinising the funnel plots, there was no indication of publication bias despite the small sample of studies included in each category. Funnel plots for positive mental and negative mental health outcomes are portrayed in Figure 6 and Figure 7, respectively. Trim-and-fill analyses were conducted to further investigate potential publication bias due to small study effects and between study heterogeneity (Duval & Tweedie, 2000). However, trim-and-fill analysis results did not recommend imputations for either category again suggesting that study effects may not be impacted by publication bias (Shi & Lin, 2019).

#### Sensitivity analysis

**Figure 8.**
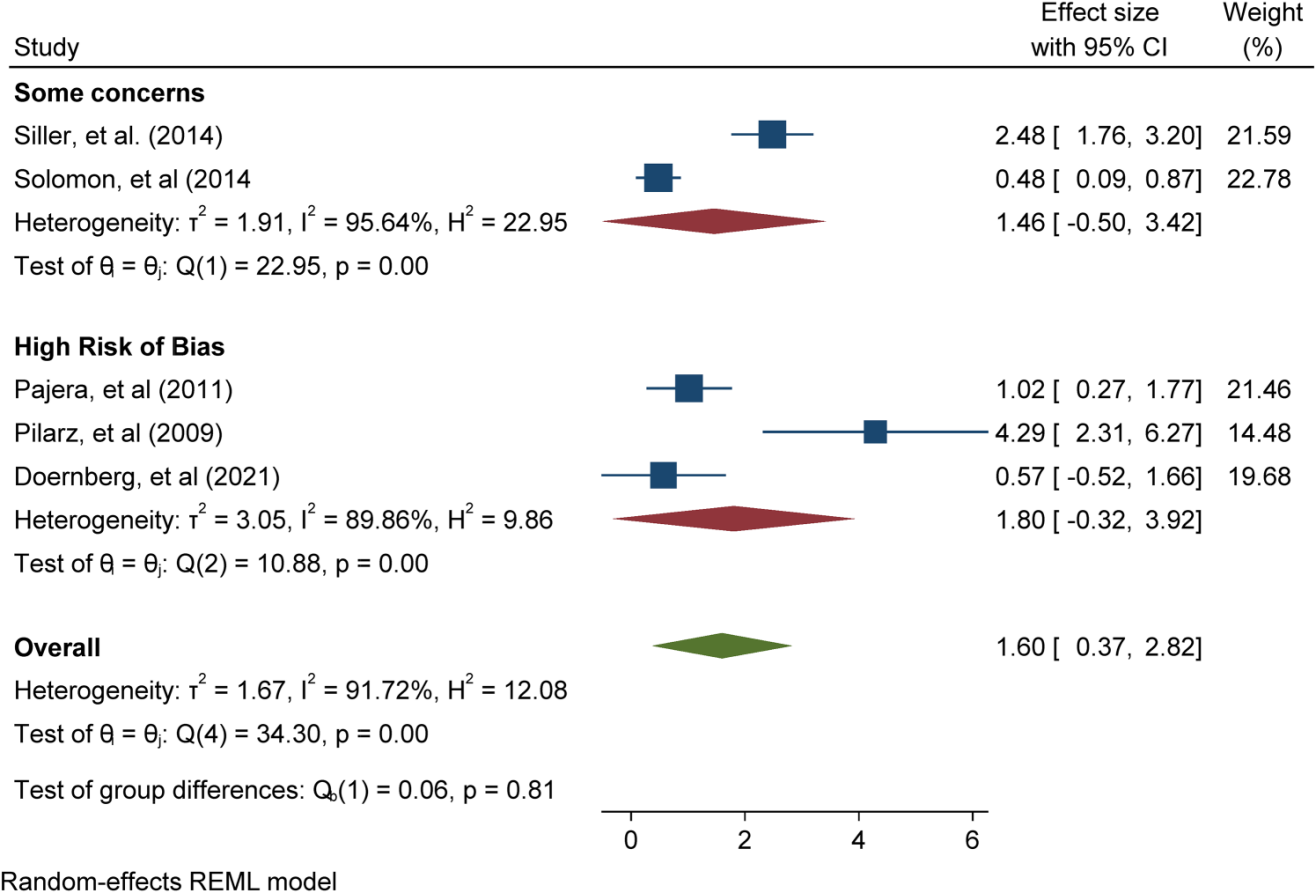
Sensitivity Analysis: Positive Mental Health by Risk of Bias Appraisal.

Sensitivity analyses were used to check if the findings for positive and negative mental health were robust to factors relating to the research design or risk of bias. With the five positive mental health studies, classification by research design comprised 4 RCTs and 1 QE and 2 studies with high risk of bias and 3 studies where there were some concerns about risk of bias. When the meta-analysis is repeated excluding the QE study, the resulting pooled effect size is slightly smaller but remains positive: 1.14 (95% CI [.22, 2.07], p  =  .02) with significant study heterogeneity Q(3)  =  23.42, p < .001, and high proportional variance I²  =  86.20%. Sensitivity analysis factoring risk of bias results using sub-group analysis (see Figure 8) indicated there was no significant difference in intervention effects depending on the risk of bias rating of studies addressing positive mental health (p  =  .81).

**Figure 9.**
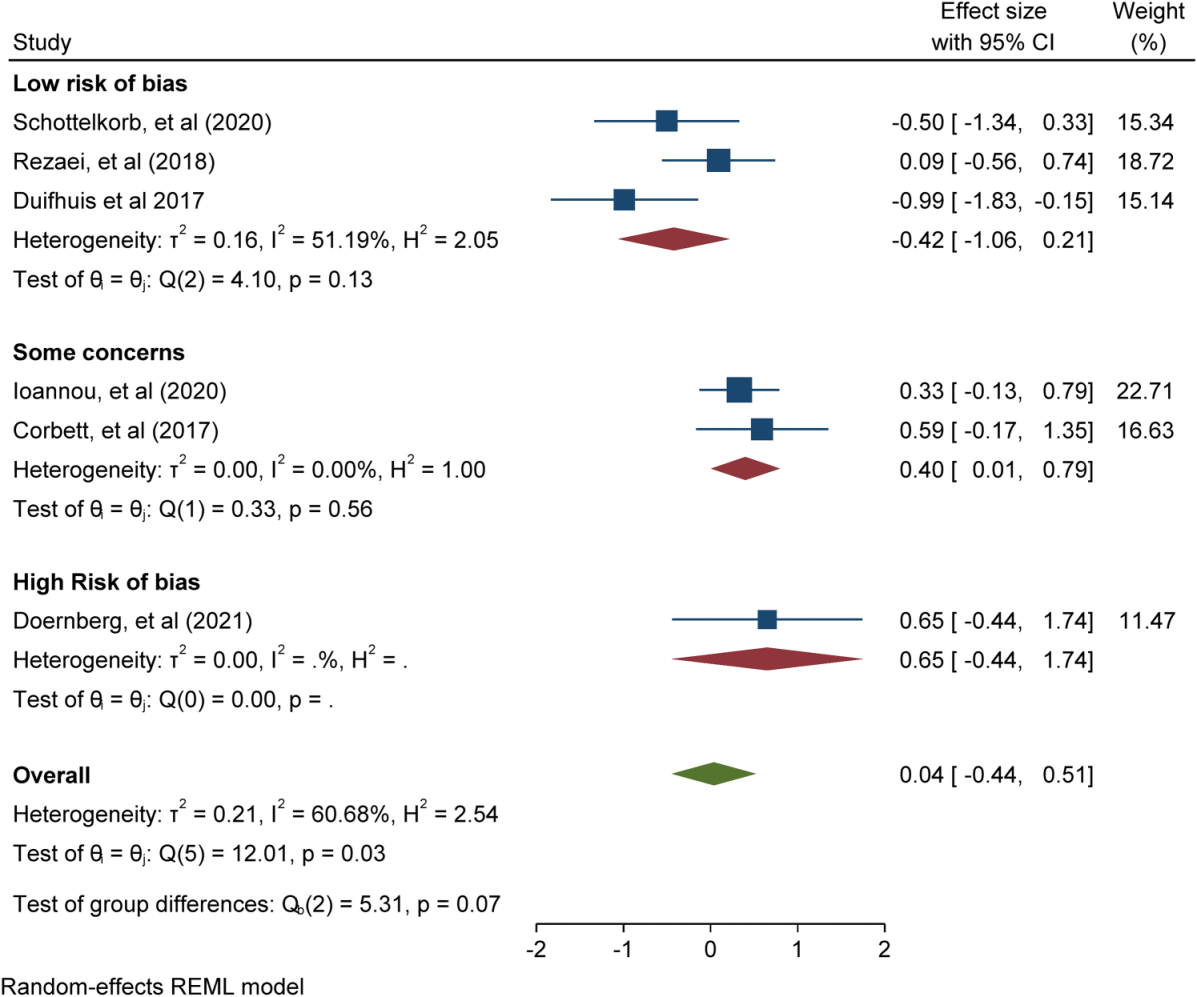
Sensitivity Analysis: Negative Mental Health by Risk of Bias Appraisal.

A similar check was done with the six studies assessing negative mental health for research design - RCT (n  =  5) and QE (n  =  1) and risk of bias - high risk of bias (n  =  1), some concerns (n  =  2), and low risk of bias (n  =  3). In this case, when the meta-analysis is repeated excluding the QE study, the intervention effect is positive but remains non-significant : .24 (95% CI [-.06, .53 ], p  =  .12) with no indication of heterogeneity among the studies Q(4)  =  4.80, p  =  .31, and proportional variance I²  =  0%. The results of the sensitivity analysis by the risk of bias results using sub-group analysis (see Figure 9) revealed no significant difference in intervention effects depending on the risk of bias rating of studies addressing negative mental health (p  =  .07).

## Discussion

A headline finding from this systematic review is the paucity of experimental work using play-based interventions to address the mental health needs of children with ASD and DLD. Only ten studies met the inclusion criteria after screening the extant literature from the past twenty years and these were reportedly undertaken only in the past twelve years. All of the studies were conducted with children with ASD; thus emphasising the existence of a gap within the play-based and mental health experimental literature for children with DLD. A recent review to identify the efficacy of support for treatment of DLD did not identify any named play-based intervention, although several studies reported delivering activities in a playful manner or via the use of computer games, storytelling, etc. (Rinaldi et al., 2021). In comparison, the use of play-based interventions with children with ASD is ubiquitous except there are fewer experimental designs that explicitly assess mental health outcomes.

### Positive mental health outcomes

The meta-analysis results support the efficacy of play-based interventions to address the positive mental health needs of children with ASD (Cohen's d  =  1.60). There were five studies grouped as positive mental health in the meta-analysis which used four different play-based interventions and assessed three different mental health outcomes. Specifically, outcomes assessed related to emotional development from a DIR/Floortime intervention (Pilarz, 2009 & Pajareya & Nopmaneejumruslers, 2011); attachment from a Focused Playtime Intervention and the PLAY Project Home Consultation, (Siller et al., 2014 & Solomon et al., 2014, respectively); and positive affect from a Pretend Play Intervention (Doernberg et al., 2021). The five individual studies all reported significant positive intervention effects and the play-based interventions, although different, were grouped as a common dependent variable.

DIR/Floortime interventions are explicitly designed to focus on emotional development and emotion building. DIR/Floortime measures changes in the ASD child's capacity for shared attention, the ability to form and have warm, intimate relationships and the ability to initiate using intentioned actions and social engagements that lead to spontaneous communication (Hess, 2012). The PLAY Project, also based on DIR/Floortime, was developed as a programme to train child development professionals to coach parents, typically at home, to help their child with ASD make developmental gains through play-based interactions (Solomon et al., 2014). Given its broader focus, the PLAY project includes other related outcomes measures like attachment (Solomon et al., 2014). Alternatively, validated interventions like Focused Playtime Intervention and the Pretend Play Intervention, generally facilitates capacity within families to meet the needs of their children by supporting social communication, play-skills, etc. and similarly assesses mental health outcomes like attachment (Siller et al., 2014; Doernberg et al., 2021).

### Negative mental health outcomes

The overall non-significant effect of negative mental health outcomes (Cohen's d  =  .04) from play-based interventions raises an important query about what factors may contribute to results different from positive mental health. Six studies were identified which used four different play-based interventions and six different measures of mental health. The outcomes related to anxiety from Sense Theatre interventions (Corbett et al., 2017; Ioannou et al., 2020); internalising and externalising behaviour using PRT and Child Centred Play Therapy (Duifhuis et al., 2017; Schottelkorb et al., 2020, respectively); irritability & hyperactivity also using PRT (Rezaei et al., 2018); and negative affect from the Pretend Play intervention by Doernberg, et al. (2021), which is the only study which included both a positive and negative measure of mental health.

The Sense Theatre is an expressive intervention that uses peer mediation with typically developing peers of ASD children to teach adaptive social behaviour. Sense theatre tries to counter negative social experiences that cause increased anxiety and stress that may lead to avoidance of social play (Ioannou et al., 2020). Both Corbett et al. (2017) and Ioannou et al. (2020) studies reported that participants receiving SENSE intervention showed significant intervention group effects on trait anxiety but not state anxiety. Child-centred Play Therapy, is another individualised mental health intervention, designed to use the relationship between therapist and child to reduce relational or physically harmful ways of interacting, as well as increasing their sense of self-responsibility toward behaviour (Ray et al., 2012). Externalising behaviour significantly decreased for participants in the Child-centered Play Therapy group at the end of the intervention but there was not a significant effect by group (Schottelkorb et al., 2020). PRT is another method used to reduce behavioural problems of children with autism and improve communication and social interaction (Rezai, et al., 2018). After using the PRT, Duifhuis et al., (2017) measured internalising and externalising behaviour as a secondary outcome and found the difference between the treatment and control group was not significant. Similarly, Rezai et al. (2018) reported there was no significant difference in behaviours relating to irritability and hyperactivity for the treatment and control group post intervention. The Pretend play intervention by Doernberg et al. (2021) reported that intervention and control groups were not dissimilar in their capacity to identify negative affect emotions.

### Intervention characteristics

As pointed out by Gibson et al. (2021), in their scoping review of play-based interventions with two- to eight-year-old children with ASD, research in this field is heterogeneous. The diversity of play-based approaches included means it is not possible to conclude that any one play-based intervention is more effective in this review. There is little detail on the extent of play engagement beyond identifying activities as including either role-play, pretend-play, toy-play, etc. The person delivering the intervention has a key role and is typically a play partner to the ASD child or facilitates training to mediate play between the ASD child and their parents or peers. The sample disproportionately represented boys (82%) and interventions were more frequently implemented with participants younger than twelve (n  =  7). The study settings were between home, school, and outside school, and durations of interventions were between one-hundred to two-hundred and forty minutes.

### Evaluation of evidence

We know that social, communication, and or language difficulties can have an adverse impact on the mental health of individuals at all ages of development (Conti-Ramsden et al., 2019; Hudson et al., 2019). The contrasting results for positive and negative mental health is particularly interesting and raises an important query about whether there are some types of [mental] outcomes that play-based interventions may not be suited to address. The limited data do not allow firm conclusions to be drawn. For instance, some play-based interventions are well-established, standardised programmes, but reported non-significant intervention effects on mental health outcomes, e.g. irritability or internalising/externalising behaviour, (Duifhuis et al., 2017; Rezaei et al., 2018). In response, we may need a theory that elucidates the characteristics of play-based interventions for supporting mental health outcomes in samples of young persons with social, communication and or language impairments and intervention studies that treat mental health as primary outcomes to minimise the influence of confounds.

We acknowledge that the studies in the review focused on children with ASD but play-based interventions have a well-established history of being used with diverse samples e.g., learning disabilities and is a popular strategy for directly addressing psychosocial problems in children (Genç et al., 2021). Interventions with DLD are necessary because children with DLD may have different mental health profiles from children with ASD. We expect that our dichotomous categorisation of positive and negative mental health may change to reflect more nuance and specific aspects of mental health once research in this field becomes less disparate.

### Strengths and limitations of the review

To minimise the introduction of potential sources of bias into the systematic review, a rigorous selection process was followed which involved pre-specified eligibility criteria, a comprehensive search of electronic databases, double screening of titles and abstracts, consensus discussions between study authors to resolve disagreements, and independent double data extraction by two pairs of study authors. In the end, there was agreement among all authors on the papers included in the review. As indicated at the beginning of the methods section, additional outcomes specific to social communication, and language were part of the literature screening but are not addressed in this paper. The risk of bias from the level of individual studies can also influence the overall meta-analysis results (Higgins et al., 2021); hence studies were appraised by two pairs of authors. About seven studies did not meet the low risk of bias threshold. From the risk of bias activity undertaken for each study, the most common issues flagged had to do with allocation concealment (6 studies) for RCTs and pre-specifying data analysis plans (5 studies), applicable to both RCTs and QEs.

### Implications and recommendations

A key implication of this work is that it raises awareness among researchers, clinicians, and practitioners to consider mental health outcomes when using play-based interventions. There is an imperative to better understand the benefits and possible drawbacks of using play-based interventions to support the mental health of individuals with social, communication, and or language impairments so stakeholders can make informed choices about treatment. We hope this review highlights the need to prioritise research investigating the efficacy of play-based interventions to support the mental health of children with SLI. It is not only important to embark on more experimental work but it is also imperative that new research strive to meet gold standards in experimental designs. The specific examples of positive mental health in the context of this review are limited to positive affect, attachment, and emotional functioning. Therefore, evidence for other plausible positive mental health variables like happiness, well-being, life satisfaction, etc. remain lacking. There is a distinct need for newer research particularly RCTs and QEs to discriminate between the diverse range of play-based interventions and their potential effect across the range of mental health outcomes - positive and negative, that exists. Furthermore, the technique of using play as a medium of engagement in interventions with children with ASD or DLD, means play cannot be used as a dependent variable in RCTs. Thus, it becomes challenging to fully assess the efficacy of play in such treatments. Gibson et al. (2021) recommends conceptualising the role of play in play-based interventions using the broad categories of “context”, “component”, or a “key mechanism” to help practitioners reflect on their views and practices regarding play. There is also still limited understanding of the intensity required to optimise intervention effects in this field.

## Conclusion

There were few published high quality RCTs or QEs investigating play-based interventions for mental health in children with ASD or DLD in the last twenty years. The few studies identified investigated play-based interventions in children with ASD and showed a beneficial effect on positive mental health but not negative mental health. This review makes a valuable contribution in presenting the state of evidence [or lack thereof] addressing the mental health needs of children with ASD or DLD.

## Notes

1. see DSM-V for further details about diagnosable mental health conditions.

2. Original source of Cochrane risk of bias tools sourced at https://www.riskofbias.info/.

3. Inaccessible papers were either because it was no longer available online or being behind a paywall, etc.

## Appendix A. Boolean Search Strings

[Table table6-23969415211073118]

**Table table6-23969415211073118:**

Play (Title) AND “language disorder” (Title & Abstract & Keyword)
Play (Title) AND “language impairment” (Title & Abstract)
Play (Title) AND “language delay” (Title & Abstract)
Play (Title) AND “language disorder” (Title & Abstract)
Play (Title) AND “speech and language” (Title & Abstract)
Play (Title) AND “speech and communication” (Title & Abstract)
Play (Title) AND “specific language impairment” (Title & Abstract)
Play (Title) AND “communication need” (Title & Abstract)
Play (Title) AND “Slow talk” (Title & Abstract)
Play (Title) AND “developmental language disorder” (Title & Abstract)
Play (Title) AND Autism (Title & Abstract)
Play (Title) AND Austistic (Title & Abstract)
Play (Title) AND pervasive develop (Title & Abstract & Field)
Play (Title) AND PDP (Title & Abstract)
Play (Title) AND Asperger (Title & Abstract & Field)
Play (Title) AND “SLI” (Title & Abstract & Full Text)
JASPER ((Joint Attention, Symbolic Play, Engagement & Regulation) (Title) & Autism
ASAP (Advancing Social-communication And Play) (Title)
DIR: Floortime (Title)
CCPT (Child-Centered Play Therapy) (Title) AND Autism (Title & Abstract & Full Text)
Filial therapy (Title)
Lego (Title) AND Autism (Title & Abstract & Full Text)
Lego (Title) AND Language (Title & Abstract & Full Text)
Lego (Title) AND speech (Title & Abstract & Full Text)
Lego (Title) AND communication (Title & Abstract)
Canine-assisted play (Title)
Theraplay (Title)
Jungian Play (Title)
Pivotal Response Treatment (PRT) (Title)

## Appendix B. Risk-of-Bias Tool (extracted from Cochrane RoB 2 and Cochrane RoB1)

[Table table7-23969415211073118]

**Table table7-23969415211073118:**

Domain 1: Risk of bias arising from the randomisation process		
Questions	Comments	Response
1.1 Was the allocation sequence random?		Y / PY / PN / N / NI
1.2 Was the allocation sequence concealed until participants were enrolled and assigned to interventions?		Y / PY / PN / N / NI
QE relevant questions - Risk of bias due to confounding		
1.3 Is there potential for confounding of the effect of intervention in this study? e.g allocations using baseline equivalents, appropriate statistical analysis of difference on pre-tests, etc.		Y / PY / PN / N / NI
1.4. Was selection of participants into the study (or into the analysis) based on participant characteristics observed after the start of intervention?		Y / PY / PN / N / NI
Risk-of-bias judgement		Low / High / Some concerns
Optional: What is the overall predicted direction of bias for this outcome?		NA / Favours experimental / Favours comparator / Towards null /Away from null / Unpredictable
Domain 2: Risk of bias due to deviations from the intended interventions (effect of assignment to intervention)		
2.1. Were participants aware of their assigned intervention during the trial?		Y / PY / PN / N / NI
2.2. Were carers and people delivering the interventions aware of participants’ assigned intervention during the trial?		Y / PY / PN / N / NI
QE relevant questions		
2.3Was the intervention clearly defined		Y / PY / PN / N / NI
2.4 Was the information used to define intervention groups recorded at the start of the intervention?		Y / PY / PN / N / NI
Risk-of-bias judgement		Low / High / Some concerns
Optional: What is the overall predicted direction of bias for this outcome?		NA / Favours experimental / Favours comparator / Towards null /Away from null / Unpredictable
Domain 3: Missing outcome data		
3.1 Were data for this outcome available for all, or nearly all, participants randomised?		Y / PY / PN / N / NI
3.2 Is there evidence that the result was not biased by missing outcome data?		Y / PY / PN / N / NI
Risk-of-bias judgement		Low / High / Some concerns
Optional: What is the overall predicted direction of bias for this outcome?		NA / Favours experimental / Favours comparator / Towards null /Away from null / Unpredictable
Domain 4: Risk of bias in measurement of the outcome		
4.1 Was the method of measuring the outcome inappropriate?		Y / PY / PN / N / NI
Risk-of-bias judgement		Low / High / Some concerns
Optional: What is the overall predicted direction of bias for this outcome?		NA / Favours experimental / Favours comparator / Towards null /Away from null / Unpredictable
Domain 5: Risk of bias in selection of the reported result		
5.1 Were the data that produced this result analysed in accordance with a pre-specified analysis plan that was finalised before unblinded outcome data were available for analysis?		Y / PY / PN / N / NI
5.2 Is the numerical result being assessed likely to have been selected, on the basis of the results, from…		Y / PY / PN / N / NI
5.3 Did they have a priori protocol?		Yes No Not clear
Risk-of-bias judgement		Low / High / Some concerns
Optional: What is the overall predicted direction of bias for this outcome?		NA / Favours experimental / Favours comparator / Towards null /Away from null / Unpredictable

**(Y)** Yes, **(PY)** Probably Yes, **(PN)** Probably No, **(N)** No, **(NI)** No information.

## Appendix

[Table table8-23969415211073118]

**Appendix C. table8-23969415211073118:** Database Screening Results By Search Terms

	Keywords searching results / Number of Studies Identified (N)								
Data Base	Ebscohost	Web of Science	Pubmed	SCOPUS	PsycINFO	PROQuest	Ethos	Google Scholar	
Search Terms Date of search	18/01/2021 22/01/2021	23/01/2021 26/01/2021	27/01/2021 31/01/2021	01/02/2021 03/02/2021	04/02/2021 06/02/2021	07/02/2021 10/02/2021	11/02/2021 12/02/2021	13/02/2021 14/02/2021	Total Identified
Play (Title) AND “Language” (Title & Abstract)	2160 / 321	1553 / 153	7080 / 155	617 / 54	298 / 69	620 / 58	13 / 3	Not Screened	813
Play (Title) AND “Speech” (Title & Abstract)	610 / 36	75 / 12	91 / 21	130 / 9	40 / 7	47 / 4	3 / 1	Not Screened	90
Play (Title) AND “Communication” (Title)	174 / 24	254 / 20	146 / 21	519 / 28	142 / 33	216 / 33	20 / 4	Not Screened	163
Play (Title) AND “specific Language Impairment” (Title & Abstract)	2/2	4 / 4	3 / 2	2 / 2	4 / 3	3 / 2	4 / -	Not Screened	15
Play (Title) AND “Developmental Language Disorder” (Title & Abstract)	5 / 5	2 / 2	2 / 2	3 / 2	27 / 6	-	-	Not Screened	17
Play (Title) AND Autis* (Title & Abstract)	477 / 396	370 / 321	298 / 220	504 / 307	459 / 399	146 / 39	130 / 19	Not Screened	1701
Play (Title) AND Pervasive Develop* (Title & Abstract & Field)	124 / 104	15 / 3	-	53 / 2	1 / -	-	6 / -	Not Screened	109
Play (Title) AND Asperger (Title & Abstract & Field)	19 / 8	21 / 11	6 / 3	8 / 5	3 / 2	10 / 5	12 / 1	Not Screened	35
Play (Title) AND “SLI*” (Title & Abstract & Full Text)	23 / 6	5 / 2	3 / 2	-	4 / -	-	7 / 1	Not Screened	11
Joint Attention, Symbolic Play, Engagement & Regulation (Title) AND Autis* (Title)	3 / 3	5 / 5	6 / 6	1 / 1	1 / 1	1 / 1	-	11 / 11	28
Advancing Social-communication (Title) AND Play (Title)	3 / 3	4 / 4	2 / 2	1 / 1	5 / 2	1 / 1	-	5 / 5	18
DIR: Floortime (Title)	15 / 11	26 / 9	12 / 8	12 / 4	50 / 15	10 / 7	1 / 1	105 / 13	68
Child-Centered Play Therapy (Title) AND Autis* (Title & Abstract & Full Text)	9 / 5	-	-	1 / 1	187 / 5	4 / 4	-	23 / 10	25
Filial therapy (Title)	46 / -	17 / -	4 / -	74 / 1	212 / 2	83 / -	-	3 / 2	5
Lego (Title) AND Autis* (Title & Abstract & Full Text)	47 / 20	11 / 9	9 / 7	14 / 11	30 / 12	10 / 10	8 / 6	43 / 31	106
Lego (Title) AND Language (Title & Abstract & Full Text)	145 / 4	59 / 1	4 / 1	19 / 1	40 / 3	3 / 1	-	40 / 2	13
Lego (Title) AND Speech (Title & Abstract & Full Text)	36 / -	-	-	8 / 2	24 / 1	-	3 / -	12 / -	3
Lego (Title) AND Communication (Title & Abstract)	35 / 2	61 / 3	7 / 3	86 / 19	1 / -	4 / 1	15 / 4	43 / 4	36
Canine-assisted play (Title)	1 / 1	-	-	1 / 1	2 / 2	1 / -	-	5 / 2	6
Theraplay (Title)	27 / 3	26 / 2	5 / -	28 / 5	66 / 8	19 / 1	1 / -	11 / 8	27
Jungian Play (Title)	42 / 1	-	45 / -	6 / 1	3 / -	-	-	22 / 1	3
Pivotal Response Treatment (PRT) (Title)	96 / 12	61 / 33	34 / 27	60 / 30	60 / 38	56 / 39	-	168 / 40	219
**Results**									
Number of Studies Screened	4099	2569	7755	2147	1659	1234	223	514	19995
Number of Studies Identified	625	516	384	383	561	187	39	128	2823
Records identified Through other sources									59
Total Number of Studies Carried to Abstract Screening									2882

Note. Limiter: Studies published between January 1st, 2000- January 15th, 2021.

## Appendix

[Table table9-23969415211073118]

**Appendix D. table9-23969415211073118:** Mental Health Classification of Studies

**MH Classification**	**Author**	**Study Measures Relating to Review Aims:** **Description Extracted from Individual Papers**	**Measurement Scales Selected**	**Measurement Scales excluded**
Positive Mental Health	Pajareya and Nopmaneejumruslers (2019)	**Primary Outcome** **The Functional Emotional Assessment Scale (FEAS)** (child behaviours) - to measure changes in children's functional development. A 15-min videotaped child–parent interaction was collected for each child. Each parent was asked to play with their child as they normally would at home using a standard set of toys (including symbolic, tactile and movement play materials). **Secondary Outcome** Functional Emotional Developmental Questionnaire (FEDQ) - The questionnaire was related to Greenspan's Six Functional Development Levels (FDL): 1) shared attention and regulation; 2) engagement and relating; 3) purposeful emotional interaction; 4) social problem solving; 5) creating ideas; and 6) thinking logically. The difference between the increments determined the clinical progression.	**FEAS** scoring reported did not include sub-scales so the summary score is used in the meta-analysis. The score is interpreted as having positive directionality **FEDQ** scoring reported did not include sub-scales so the summary score is used in the meta-analysis. The score is interpreted as having positive directionality	No sub-scales were excluded No sub-scales were excluded
Positive Mental Health	Solomon et al. (2014)	**Primary Outcome** **The Functional Emotional Assessment Scale** – is a video assessment of a child's interactional/ social functioning. The FEAS has 6 sections and 34 items based on Greenspan's 6 functional developmental levels (FDLs), 33 which progress from simple attention (FDL 1) and engagement (FDL 2) to 2-way purposeful reciprocal exchanges (FDL 3), to problem solving gestures (FDL 4), and then to the consistent use of words (FDL 5) leading to rich *pretend play, emotional thinking, and complex interaction (FDL 6).* Items are rated as 0 (not at all or very brief), 1 (present some of the time, observed several times), or 2 (consistently present, observed many times). Ratings were summed to compute scores. *Higher raw scale scores indicate greater social–emotional development.* Items are rated as 0 (not at all or very brief), 1 (present some of the time, observed several times), or 2 (consistently present, observed many times). Ratings were summed to compute scores. Higher raw scale scores indicate greater social–emotional development. Parent child free play with toys in the home was video recorded for 15 min at pre- and post-assessment and coded by raters blind to group allocation and assessment time. **Child Behaviour Rating Scale (CBRS)** - The CBRS is composed of 7 items, which assesses 2 interactive style dimensions for children: Attention (4 items, a 5 .88 at baseline and .89 at follow-up) and Initiation (3 items, a 5 .70 at baseline and .83 at follow-up).	**FEAS** scoring reported did not include sub-scales so the summary score is used in the meta-analysis. The score is interpreted as having positive directionality. **CBRS** measure selected and both subscales are used: Attention Initiation	No sub-scales were excluded No sub-scales were excluded
Positive Mental Health	Pilarz (2009)	**Primary Outcome** **The Functional Emotional Assessment Scale (FEAS)** - The FEAS assesses children whose developmental age is between 7 months and 4 years, who are at risk or have problems in the development of attachment, regulation, social engagement, play interactions and emotional functioning. The FEAS focuses on the dynamic interaction between caregiver-child. The FEAS provides a formal system of coding child and caregiver's behaviours using the six developmental levels of the DIR/Floortime Model.	**FEAS** measure selected but only the ‘total sub-scale’ is used in the meta-analysis. The score is interpreted as having positive directionality.	FEAS subscales excluded: Subscale 1 - Attachment Subscale 2 - Regulation Subscale 3 - Social engagement Subscale 4 - Play interactions Subscale 5 - Emotional functioning
Positive Mental Health Outcomes	Doernberg, et al. (2021)	**Primary Outcome** **The Kusche Affective Inventory Revised (KAI-R)** - a child interview-based measure used to directly evaluate children's emotional understanding abilities. Two sections: *1) their ability to generate lists of feelings and describe emotional experiences of simple and complex emotions, while defining more complex emotions, and 2. their ability to understand the emotional states of self and other).* (For the emotional experience section, children were asked to generate a list of as many positive and negative feelings that they could without a time limit, and up to three prompts of “Any more?” by the researcher. A total frequency score was then calculated for positive emotions, negative emotions, and total number of emotions. *Responses were coded only if they qualified as valid positive/negative emotions per the manual (Greenberg* et al., *1995)*. Each child was next asked to provide examples of times when he/she had felt 10 specific emotions, ranging from simple to complex: happy, sad, angry, scared, love, proud, guilty, jealous, anxious, and lonely (e.g., “Tell me about a time when you felt happy?”). If a child had difficulty providing an example for a particular emotion, he/she was asked to give an example of a time when someone else felt that emotion (Greenberg et al., 1995). Responses were coded on a 4-point developmental scale according to the manual (Greenberg et al., 1995). A sum score was calculated for the child's ability to appropriately describe an emotional experience (sum of all coded responses across the 10 different emotions based on the 4-point scale). Additionally, a sum score was calculated for the child's quality in defining a more complex emotion (sum of all coded responses across the five latter complex emotions— proud, guilty, jealous, anxious, and lonely—based on a 0–2 scale from Greenberg et al., 1995). For the understanding of emotional states of self and other section of the KAI-R, children were asked to provide cues used to recognise emotional states in themselves and others. The children were asked about five emotional states: happy, sad, scared, angry, and love (e.g., for self: “How do you know when you are feeling happy?”, for other: “How do you know when other people are feeling happy?). The responses were coded in accordance with the cognitive developmental framework of Carroll and Steward (1984), on a 4-point developmental scale according to the manual (Greenberg et al., 1995). The scores for each of the five emotions for self and other were summed to provide two total scores of understanding of emotional states of self, and understanding of emotional states of other.	**KAIR** measure selected but only the following sub-scales are used: Total positive affect (coded as positive mental health) This scale consists of counts of positive feelings and is interpreted as having positive directionality.	KAIR excluded Scales: Total affect Total appropriate Total quality Total understanding of self Total understanding of other
Negative Mental Health	Doernberg, et al. (2021)	**Primary Outcome** The Kusche Affective Inventory Revised (KAI-R) -	**KAIR** measure selected but only the following was sub-scales used: Total negative affect (coded as negative mental health). The scale consists of counts of negative feelings and is interpreted as having positive directionality.	KAIR excluded Scales: Total affect Total appropriate Total quality Total understanding of self Total understanding of other
Negative Mental Health	Corbett, et al. (2017)	**Primary Outcome** STAI-C. The STAI-C is a questionnaire - The scale is based on the original *STAI*, modified for children, and has been used to measure anxiety in numerous previous intervention studies. The inventory measures state (current) and trait (persistent) anxiety. Alpha reliability of the *STAI-C* is high, ranging from 0.78 to 0.91 (Muris 2002). Test–retest reliability for *STAI-C Trait* is higher (0.65–0.71) than the test–retest reliability for *STAI-C State* (0.31–0.41), but this is valid, as the *STAI-C State* is intended to measure a transient state (Julian, 2011). Furthermore, *STAI-C* effectively distinguishes between those with and without anxiety disorders (Seligman et al., 2004) and correlates with other measures of anxiety (Spielberger & Edwards, , 1973).	**STAIC** measure selected and both subscales are used: Trait anxiety State anxiety This scale expresses negative emotions and is interpreted as having negative directionality or lower scores imply less anxiety	No subscales were excluded
Negative Mental Health	Ioannou, et al., (2020)	**Primary Outcome** State-Trait Anxiety Inventory for Children - The STAIC is a self-report questionnaire aimed to measure both State (current) and Trait (enduring) anxiety (Spielberger et al., 1983). It has been utilised among both TD youth (e.g., Muris & Merckelbach, 1998) and youth with ASD (Lanni et al., 2012; Park et al., 2013; Simon & Corbett, 2013). Alpha reliability ranges from 0.78 to 0.91; test–retest reliability for the STAIC-Trait is 0.65–0.71 (Julian, 2011). Participants in the EXP and WLC groups were administered the STAIC after the PIP.	**STAIC** measure selected and both subscales are used: Trait anxiety State anxiety This scale expresses negative emotions and is interpreted as having negative directionality or lower scores imply less anxiety	No subscales were excluded
Negative Mental Health	Rezaei et al. (2018)	**Primary Outcome** **Aberrant Behavior Checklist (ABC):** The checklist includes 58 questions designed to assess *the presence and severity of maladaptive behaviors in people with developmental disabilities*. This tool evaluates five categories of behavioural disorders, three of which are the main defects in autism: lethargy, stereotypical behaviour, and inappropriate speech, with the other two being related to *hyperactivity/restlessness and irritability*.	**ABC** measure selected but only the following subscales were used: hyperactivity/restlessness Irritability The scale expresses negative emotions and is interpreted as having negative directionality or lower score imply less hyperactivity/restlessness and irritability,	ABC subscales excluded: Lethargy Stereotypical behaviour Inappropriate speech
Umar to decide the categorisation of the attachment outcome variable (According to the attachment scales that the authors used)	Siller et al., (2014)	**Primary Outcome** **The Proximity and Contact Seeking Behaviors (PCSB) Scale** “*The PCSB evaluates the intensity of a child's effort to regain contact with, or proximity to, their mother. Higher PCSB scores indicate that the child took initiative in achieving contact, where lower scores indicate the child made no effort to make contact with their mother (p.1725)”*. **The Avoidant Behaviors (AB) Scale** “*The AB evaluates the intensity and duration of the child's avoidance toward their mother. Lowest AB scores indicate that the child did not greet his/her mother upon reunion despite the mother's attempts at interaction, where higher scores indicate that the child did not display avoidant behaviors toward his/her mother*. *Please note that both measures were scored so that higher numbers represent stronger signs of attachment (p.1725)”*. Observed Attachment Behaviours (mean of PCSB and AB) The authors created a global measure of observed attachment behaviours, which was computed as the average of children's PCSB and AB scores. Higher means represent stronger signs of attachment. **Maternal Perceptions of Child Attachment questionnaire (MPCA;Hoppes and Harris 1990).** “*This parent-report measure consists of 23 items rated on a 5-point rating scale, ranging* *from frequently (1) to never (5). High scores indicate maternal perceptions of strong child attachment (p.1725)”.*	**PCSB** measure did not include sub-scales so the total score is used in the meta-analysis. PCSB total score is interpreted as higher numbers represent stronger signs of attachment. **AB** measure did not include sub-scales so the total score is used in the meta-analysis. AB total score is interpreted as higher numbers represent stronger signs of attachment. **Observed Attachment Behaviour** measure did not include any subscales as it is created as the sum of PCSB and AB measures. Therefore, the total score in this scale is interpreted as higher means represent stronger signs of attachment. **MPCA** measure did not include sub-scales so the total score is used in the meta-analysis. MPCA total score is interpreted as higher numbers represent stronger signs of attachment.	No subscales were excluded
Negative Mental Health	Schottelkorb et al. (2020)	**Primary Outcome** **Child Behavior Checklist (CBCL)** - The CBCLs for children ages 1 to 5 and 6 to 18 are instruments of the Achenbach System of Empirically Based Assessment. *Both measures are used for examining emotional and behavioral problems as well as adaptive functioning of children as rated by parents/guardians*. In our study, parents rated the items on the CBCL on a 3-point Likert-type scale ranging from 0 (*not true*) to 2 (*very true or often true*). *The instruments are designed to evaluate child behaviors across three domains—externalizing, internalizing, and total behavior problems—using various subscales*. Due to the varied ages of participants in this study, the two different CBCL versions were used. *Because the two versions offer differing subscales, we focused on comparing pre-post data from those subscales that were consistent across the two types: Attention Problems, Aggressive Behavior, and Externalizing Problems. Scoring procedures for the CBCL involve calculating T scores and percentiles for each subscale. T scores between 60 and 63 are considered borderline, suggesting an area of concern, and T scores higher than 63 are considered clinical*. Both versions of the CBCL (1–5 and 6–18) are reliable (test-retest coefficients between .68 and .92) and valid for identifying individuals with internalizing and externalising behaviours (Achenbach & Rescorla, 2001).	**CBCL** measure selected but only the following subscale was used: externalising problems The scale expresses negative emotions and is interpreted as having negative directionality or lower score imply less externalising problems	CBCL subscales excluded: Attention Problems Aggressive Behaviour
Negative Mental Health	Duifhuis et al. (2017)	**Secondary Outcome** **Child Behavior Checklist (CBCL)** - behavioral problems were measured using parents’ ratings on the Dutch translation of the Child Behavior Checklist (CBCL). This is a widely used standardised questionnaire for those aged either 1 − 5 or 6–18 years (Achenbach & Rescorla 2000). *The CBCL is a parent rating scale which measures children's general problem behavior and internalizing and externalizing behavior, while more specific problem behaviors are assessed with supplementary scales*. *In the analyses of this study, three major scales in the CBCL were focused on: The total scale, externalizing behavior, and internalizing behavior*. Cut-off scores for clinically elevated symptoms are based on T-scores ≥ 68 (Achenbach ∧ Rescorla 2000). Internal consistencies (Cronbach's Alpha) for the Dutch version were found to be >0.90 (Verhulst et al., 1996).	**CBCL** measure selected and the following subscales were used: Externalising behaviour Internalising behaviour High scores are indicators of negative mental health so the scale is interpreted as having positive directionality	CBCL subscales excluded: Total scale
